# Traces of business cycles in credit-rating migrations

**DOI:** 10.1371/journal.pone.0175911

**Published:** 2017-04-20

**Authors:** Dmitri Boreiko, Serguei Kaniovski, Yuri Kaniovski, Georg Pflug

**Affiliations:** 1 Faculty of Economics and Management, Free University of Bozen-Bolzano, Bolzano, Italy; 2 Macroeconomics and European Economic Policy Research Group, Austrian Institute for Economic Research (WIFO), Vienna, Austria; 3 Department of Statistics and Decision Support Systems, University of Vienna, Vienna, Austria; Universidad Veracruzana, MEXICO

## Abstract

Using migration data of a rating agency, this paper attempts to quantify the impact of macroeconomic conditions on credit-rating migrations. The migrations are modeled as a coupled Markov chain, where the macroeconomic factors are represented by unobserved tendency variables. In the simplest case, these binary random variables are static and credit-class-specific. A generalization treats tendency variables evolving as a time-homogeneous Markov chain. A more detailed analysis assumes a tendency variable for every combination of a credit class and an industry. The models are tested on a Standard and Poor’s (S&P’s) dataset. Parameters are estimated by the maximum likelihood method. According to the estimates, the investment-grade financial institutions evolve independently of the rest of the economy represented by the data. This might be an evidence of implicit too-big-to-fail bail-out guarantee policies of the regulatory authorities.

## Introduction

Analyzing historical credit ratings, this paper aims at probabilistically characterizing both their observable and hidden drivers by studying long-run regularities and instantaneous affects related to business cycles.

A class of credit-risk models treats credit-rating migrations as trajectories of a time-homogeneous Markov chain. The corresponding Markovian matrix *P* is called the historical or the unconditional migration matrix. The assumption of time-homogeneity implies that, considering a longer period of observation, more reliable estimates of *P* are obtained. This fact explains the meaning of the adjective historical. The matrix reflects the average or typical economic situation for the period. The word unconditional refers to the fact that the probabilities do not depend on the macroeconomic conditions. The models entail no explicit mechanism for imposing dependence among migrations. The main principles of the approach were developed during the last decade of the 20-th century [1], [2] and [3].

In order to enhance the explanatory power of credit-risk models, macroeconomic factors were introduced into consideration. (More frequent defaults during a recession is a stylized fact evidencing that credit-rating migrations are influenced by macroeconomic factors.) Being affected by the same macroeconomic factors, migrations turn out to be dependent under this modeling approach. The corresponding migration probabilities are referred to as conditional. Different macroeconomic factors, risk models incorporating them and related estimation techniques are considered in [4], [5], [6], [7], [8], [9], [10], [11]. Some of these models entail a regime-switching matrix. This matrix and its steady-state distribution are statistical characteristics of a business cycle. For example, analyzing data of the National Bureau of Economic Research (NBER), Bangia et al. conclude that: “While the 1981–1998 regime-switching matrix implies that on average 17.8% of all quarters are contraction quarters, the 1959–1998 regime-switching matrix predicts 20.9% of all quarters to be contractions, indicating that the economic development over the last 20 years has been relatively favorable. Moreover, recessions seem to be getting shorter as evidenced by the maintain probability declining from 42.4% (1959–1998) to 30.8% (1981–1998)”. ([4] p. 467) This is a quantitative characterization of business cycles in the US economy. Their quarterly 2 × 2 Markovian regime-switching matrix contains probabilities of the events that a recession (expansion) now will be followed by a recession (expansion) in the next quarter of a year. In particular, the probability that a recession is followed by a recession, the maintain probability for recessions, equals 0.308 (0.424) during 1981–1998 (1959–1998). The steady-state distribution for the period 1981–1998 (1959–1998) is (0.822, 0.178) ((0.791, 0.209)). The first (second) coordinate is the steady-state probability of a recession (expansion) quarter.

A coupling scheme was introduced in [12] as a technique for modeling of dependent credit-rating migrations. The term coupling scheme means that a migration is split into two components: an idiosyncratic and a common one. They are identically distributed. The distribution coincides with the corresponding row of a historical matrix *P*. Being involved in several migrations, a common component renders them dependent. The conditional distribution of a common component is parametrized by macroeconomic factors so that migrations towards riskier credit classes are more (less) likely under adverse (favorable) macroeconomic conditions. Migrations towards safer credit classes exhibit the opposite trend: they are more (less) likely under favorable (adverse) macroeconomic conditions. The common components whose conditional distributions are parametrized by the same macroeconomic factors are stochastically dependent. All other things equal, the impact of microeconomic factors depends upon how big is the pool of debtors who are affected by the same common component. A common component affecting all debtors from a credit class or a debtor-specific common component are the extreme situations considered in [12] and [13]. (In [13], common components are conditionally independent across debtors. In this sense, they are debtor-specific. Since the conditions are the same for all debtors belonging to a credit class, migrations of such debtors are dependent.) A common component specific for each combination of a credit class and an industry sector is considered in [14].

The economy studied here is synthetic. It is populated by companies rated by a credit agency. The analysis uses a S&P’s dataset that contains credit-rating migrations for the period 1991–2015. This economy comprises 13613 companies from the OECD (Organization for Economic Co-operation and Development) countries.

As the *first quantitative characteristic* of a business cycle, the conditional migration probabilities and their variation with respect to the unconditional ones are estimated. The variation is measured as the percentage of adjustment of upgrading and downgrading probabilities during periods of favorable and adverse macroeconomic conditions.

The *second quantitative characteristic* of a business-cycle dynamics considered here is the transition matrix of the hidden Markov chain governing this dynamics. It contains probabilities of having favorable (adverse) macroeconomic conditions in the next time instant given the macroeconomic conditions now. In other words, the most likely succession of expansions and recessions that matches a given record of credit-rating migrations is indicated. This analysis involves sub-economies formed by debtors belonging to a credit class. Rather than trying to identify periods of recession or expansion, which is difficult for such sub-economies, we focus on estimating probabilistic characteristics of such periods.

Correlations between macroeconomic factors affecting different sub-economies, is the *third quantitative characteristic* of a business cycle suggested here. In this case, a sub-economy corresponds to a combination of a credit class and an industry sector. If a correlation coefficient is close to one, the macroeconomic factors behind the two sub-economies are coherent: the sub-economies pass phases of a business cycle synchronously. The correlations close to zero imply that the factors affecting the corresponding credit classes and industries are independent. Finally, a negative correlation indicates a mismatch of the factors: an upswing in some of the sub-economies could coincide temporally with a decline in the others. Competition for the market place implying a substitution pattern is a plausible explanation of the mismatch.

## Coupling schemes and conditional migration probabilities

Consider a portfolio where debtors are non-homogeneous in their credit ratings. Let there be *M* ≥ 1 non-default credit classes. Numbering them in a descending order, let us assign 1 to the most secure assets, while the next to default credit class is indexed by *M*. Defaulted firms receive the index *M* + 1.

Within the CreditMetrics approach [2], an *M* × (*M* + 1) Markovian transition matrix *P* = (*P*_*i*,*j*_) is estimated. Its entry *P*_*i*,*j*_ equals the probability that a debtor belonging to credit class *i* at time *t* will move to credit rating *j* at time *t* + 1, *t* = 1, 2, …. That is, the credit rating of a debtor is a random variable whose distribution at *t* + 1 depends only on the credit rating at *t* and the corresponding transition probabilities do not depend upon *t*. Typically annual transitions are considered. The *M* + 1-th row of *P* is never quoted. According to our notation, it corresponds to the defaulted debtors. It is conventionally assumed that a defaulted debtor never returns to business, at least under its original name. Mathematically, this fact is characterized by the following relations: *P*_*M*+1,*j*_ = 0 for *j* = 1, 2, …, *M*, and *P*_*M*+1,*M*+1_ = 1. The first *M* of them imply that a defaulted debtor never returns to business or, equivalently, he will never receive a non-default ranking again. The last one means that the debtor remains defaulted forever. In technical terms, *M* + 1 is an absorbing state of this time-homogeneous, discrete-time Markov chain. Conceptually, there is a problem associated with withdrawn ratings. They are referred to as Not Rated (NR) and are typically ignored or analyzed separately.

Representing the average or the typical market situation for a period of time, *P* does not account for rapid changes of economic conditions. This inability to reflect the current macroeconomic situation has been criticized and different ways to improve the estimates of migration probabilities have been suggested [1], [4], [5], [9].

Coupling schemes considered in [12], [13] and [14] are re-interpreted and generalized here. We focus on the probabilistic characteristics of credit-rating migrations and on the market conditions shaping them.

Complexity of the models given next increases progressively. It renders them, on the one hand, more realistic but, on the other hand, more demanding in what concerns the necessary computational resources. In the simplest case, the macroeconomic factors are modeled as credit-class-specific, their impact is not diversified across industries and they do not evolve in time. As the next step, a static model with diversification of credit-class-specific macroeconomic factors across industries is considered. Next, the model is equipped with a Markovian macroeconomic dynamics. Finally, a static model with macroeconomic factors specific for every combination of a credit class and an industry is presented.

Let us begin with the simplest setting. Let N be the initial size of the portfolio. Assign a number n=1,2,⋯,N, to every debtor in the portfolio at time *t* = 1. Set *X*_*n*_(*t*) for the credit rating at time *t* ≥ 1 of the debtor numbered by *n*. Then *X*_*n*_(*t*) is a discrete-time Markov chain with *M* + 1 states: 1, 2, …, *M* + 1. The evolution of the whole portfolio is captured by a random process $\vec{X}\left(t\right)=\left(X_{1}\left(t\right),X_{2}\left(t\right),\ldots ,X_{N}\left(t\right)\right)$ whose components are stochastically dependent. The dependence is induced by a coupling technique.

The simplest coupling technique generates a joint distribution such that: migrations of debtors through credit classes are dependent and each of them is governed by the same Markovian matrix *P*. To describe a coupling scheme, it is enough to consider a transition from time *t* = 1 to time *t* = 2. To this end, introduce N independent in *n* random variables *ξ*_*n*_. Each of them assumes values 1, 2, …, *M* + 1. The corresponding distribution is given by the *X*_*n*_(1)-th row of *P*:

$$\begin{array}{c} P\left\{\xi_{n}=j\right\}=P_{X_{n}\left(1\right),j}. \end{array}$$

*ξ*_*n*_ is interpreted as an idiosyncratic component of the transition from *X*_*n*_(1) to *X*_*n*_(2). Its impact on the resulting move is determined by a Bernoulli random variable *δ*_*n*_ according to the formula:

$$\begin{array}{c} X_{n}\left(2\right)=\delta_{n}\xi_{n}+\left(1-\delta_{n}\right)\eta_{n}. \end{array}$$ (1)

Here, *η*_*n*_ stands for a common component in the transition from *X*_*n*_(1) to *X*_*n*_(2). Random variables *δ*_*n*_ are independent in *n*. In general, the dependence among *X*_*n*_(2) increases as the probability of success $E\delta_{n}$ of *δ*_*n*_ decreases. In an extreme case of $E\delta_{n}=1$, the variable *δ*_*n*_ is deterministic, that is *δ*_*n*_ ≡ 1. Then the credit rating migration at *t* = 1 of the debtor numbered by *n* is governed exclusively by the idiosyncratic component and, consequently, it does not depend upon migrations of the rest of the portfolio. The sets of random variables {*δ*_*n*_}, {*ξ*_*n*_} and {*η*_*n*_} are independent.

Eq (1) resembles the common factor models, where a common factor renders defaults of different debtors dependent [4], [15]. In that case, the weights determining strength of dependence are deterministic. As a consequence, relation Eq (1) defines a mixture of two distributions, while the common factor models involve a convolution of them.

Affecting distributions of common components, the macroeconomic factors render them dependent. The adjustment requires representing a macroeconomic outcome as a binary vector. Its coordinates, termed as tendency variables, modify distributions of the corresponding common components. Note that dependent common components can co-exist with idiosyncratic migrations: $E\delta_{n}$, balancing the relative impact of the two terms in the migration defined by Eq (1), determines how strong the macroeconomic factors affect this migration.

To define credit-class-specific tendency variables, consider the set of all vectors $\vec{\chi }$ with *M* coordinates, each 0 or 1. Numbering them and assigning probability *π*_*i*_ to ${\vec{\chi }}^{\left(i\right)},\;i=1,2,\ldots ,2^{M},$ we obtain a probability distribution $\vec{\pi }=\left(\pi_{1},\pi_{2},\ldots ,\pi_{2^{M}}\right)$. A tendency vector $\vec{\Pi }=\left(\Pi_{1},\cdots ,\Pi_{M}\right)$ is a random vector whose distribution is $\vec{\pi }$, $P\left\{\vec{\Pi }={\vec{\chi }}^{\left(i\right)}\right\}=\pi_{i}$. Its coordinates Π_*i*_ are termed as tendency, hidden or latent variables.

Let $\vec{\chi }=\left(\chi_{1},\ldots ,\chi_{M}\right)$ be a realization of a tendency vector. Its coordinate *χ*_*i*_ affects the evolution of debtors from credit class *i*. When *χ*_*i*_ = 1, all of the random variables *η*_*n*_, such that *X*_*n*_(1) = *i*, cannot assume the values larger than *i*. If credit class migrations of every debtor belonging to credit class *i* were governed exclusively by the corresponding *η*_*n*_, this would have meant that the credit rating of such debtors cannot worsen. For this reason, we interpret the situation when *χ*_*i*_ = 1 as a non-deteriorating or favorable tendency for them. In the same way, *χ*_*i*_ = 0 implies that all of the random variables *η*_*n*_, such that *X*_*n*_(1) = *i*, take on exclusively the values exceeding *i*. Because the credit rating worsens, it is termed as a deteriorating or an adverse tendency.

The corresponding conditional distribution,

$$\begin{array}{c} P\left\{\eta_{n}=j|\vec{\chi }\right\}=P_{X_{n}\left(1\right),j}\left(\chi_{X_{n}\left(1\right)}\right), \end{array}$$

of *η*_*n*_ is defined in the following way:

$$\begin{array}{c} P_{i,j}\left(1\right)=\{\begin{array}{cc} P_{i,j}/P_{i} & \;\text{if}\;j\leq i,\\ 0 & \;\text{if}\;j>i; \end{array}\;\text{and}\;\;P_{i,j}\left(0\right)=\{\begin{array}{cc} P_{i,j}/\left(1-P_{i}\right) & \;\text{if}\;j>i,\\ 0 & \;\text{if}\;j\leq i. \end{array} \end{array}$$ (2)

Here, *P*_*i*_ = *P*_*i*,1_ + *P*_*i*,2_ + … + *P*_*i*,*i*_. If $P\left\{\Pi_{i}=1\right\}=P_{i}$, this choice of conditional probabilities guarantees that each migration is governed by *P* unconditionally or on average. In fact, then the unconditional distribution of *η*_*n*_ coincides with the *X*_*n*_-th row of *P*.

The macroeconomic factors, represented by tendency variables, render migrations towards safer credit classes more probable under favorable macroeconomic conditions and, respectively, less probable, when the conditions are adverse. It is worthwhile to remark that the common factor models do not involve a mechanism adjusting default probabilities according to the macroeconomic conditions. The resulting effect—adjustment of transition probabilities according to macroeconomic conditions—is the same as when a mixture of Markov chains (MMC) is used [5], [7].

Having a distribution $\vec{\pi }$, coefficients of correlation between hidden variables can be evaluated. They may be interpreted in terms of macroeconomic factors affecting debtors belonging to the corresponding credit classes. In fact, the factors acting in the same direction imply coefficients of correlation close to one. A coefficient of correlation equal to zero means that the factors are independent. (Since tendency variables take on only two values, 0 and 1, zero correlation between two of them is equivalent to their independence.) If phases of a business cycle, an expansion or a contraction, propagate through the two credit classes with a delay, the coefficient of correlation may take an intermediate value, between zero and one. Finally, negative correlations, could mean that the sub-economies compete for the market place. Since creditworthiness is the only classification criteria, a sub-economy comprises all debtors belonging to a credit class. Conventionally, arguing about phases of a business cycle, economists mean the whole economy. In our terms, it is equivalent to considering a single non-default credit class. That is, *M* = 1. Contemporary economic statistic does consider sub-economies corresponding to different credit classes. In this sense, the tendency variables are referred to as non-observable or hidden.

Dependence among {*η*_*n*_} can be defined in two ways. One of them, considered in [12], assumes that a common component remains the same for all debtors belonging to a credit class and that these random variables are conditionally independent across credit classes. In formal terms, random variables *η*_*n*_ and *η*_*l*_ are conditionally on $\vec{\Pi }$ independent if *X*_*n*_(1) ≠ *X*_*l*_(1), while *η*_*n*_ = *η*_*l*_ if *X*_*n*_(1) = *X*_*l*_(1). Alternatively, common components are defined as debtor-specific in [13]. That is, *η*_*n*_ are conditionally on $\vec{\Pi }$ independent in *n*. For a fixed set of parameters, the first model should, intuitively, impose a stronger dependence among migrations than the second one. The corresponding formal argument is given in [13] and [14]. In what follows next, these models are referred to as model (scheme) one and two.

Fitting such a model to historical credit rating migration data, the Markovian matrix *P* is assumed to be given, while the distribution $\vec{\pi }$ and the probabilities of success $E\delta_{n}$ have to be estimated. Since the only classification criterion is creditworthiness, debtors belonging to a credit class are not distinguishable. Consequently, all possible probabilities of success are summarized by a 1 × *M* matrix *Q* = (*q*_1_, *q*_2_, …, *q*_*M*_). Since for each coordinate of a tendency vector two values, 0 and 1, are allowed, 2^*M*^ unknowns are required to characterize the probability distribution $\vec{\pi }$ of hidden variables. In sum, there are *M* + 2^*M*^ unknown parameters.

Relations Eqs (1) and (2) imply that transition probabilities governing credit-rating migrations are adjusted according to the macroeconomic conditions that are encoded by the corresponding hidden variables. The adjustment is scaled by the respective $E\delta_{n}$ as the estimates given next show. Let ${\hat{P}}_{i,j}\left(\cdot \right)$ be the conditional on $\vec{\Pi }$ transition probabilities governing migrations of a representative debtor. They account for both terms in Eq (1) according to the frequencies of the corresponding elementary migrations in the pool. (The adjective elementary means that neither *ξ*_*n*_, nor *δ*_*n*_ can be split.) The probabilities ${\hat{P}}_{i,j}\left(\cdot \right)$ can be interpreted as instantaneous because the historical probabilities *P*_*i*,*j*_ are modified depending upon the current macroeconomic conditions. Consider a credit class *i*. Conditional on *χ*_*i*_ = 1,

$$\begin{array}{c} {\hat{P}}_{i,j}\left(1\right)=\{\begin{array}{cc} q_{i}P_{i,j}+\left(1-q_{i}\right)\frac{P_{i,j}}{P_{i}} & \;\text{if}\;j\leq i,\\ q_{i}P_{i,j} & \;\text{if}\;j>i; \end{array} \end{array}$$

while conditional on *χ*_*i*_ = 0,

$$\begin{array}{c} {\hat{P}}_{i,j}\left(0\right)=\{\begin{array}{cc} q_{i}P_{i,j} & \;\text{if}\;j\leq i,\\ q_{i}P_{i,j}+\left(1-q_{i}\right)\frac{P_{i,j}}{1-P_{i}} & \;\text{if}\;j>i. \end{array} \end{array}$$

Here, $\vec{\chi }$ stands for a realization of $\vec{\Pi }$. These formulas show that coupling schemes belong to the point-in-time methodologies [5], [8]. Consider the percentage of variation Δ_*i*,*j*_(*χ*_*i*_) of the historical probability *P*_*i*,*j*_ depending upon macroeconomic conditions:

$$\begin{array}{c} \Delta_{i,j}\left(\chi_{i}\right)=\frac{{\hat{P}}_{i,j}\left(\chi_{i}\right)-P_{i,j}}{P_{i,j}}100. \end{array}$$

For *j* ≤ *i*(*j* > *i*) this is an upgrading (a downgrading) probability. The corresponding formulas are summarized in Table 1. In a credit class *i*, the percentage of variation is scaled by the multiplier 1 − *q*_*i*_. Note that the same multiplier characterizes dependence of credit rating migrations in this credit class on the rest of the portfolio. In particular, the extreme value *q*_*i*_ = 1 implies no effect of the macroeconomic factors on migrations in the credit class *i* as well as independence of these migrations of what happens in the remaining credit classes. In general, the multipliers $\left(1-q_{i}\right)\frac{1-P_{i}}{P_{i}}$ and $\left(1-q_{i}\right)\frac{P_{i}}{1-P_{i}}$ can exceed 1 or fall below this value. However, $\left(1-q_{i}\right)\frac{1-P_{i}}{P_{i}}<\left(1-q_{i}\right)$ and $\left(1-q_{i}\right)\frac{P_{i}}{1-P_{i}}>\left(1-q_{i}\right)$ when *P*_*i*_ > 1/2. Since for all known credit classes typically *P*_*i*_ > 1/2, it follows that, for this kind of models, the *macroeconomic factors influence less an upgrading probability than a downgrading one* and *the latter is affected stronger by the adverse than by the favorable macroeconomic factors*. This is consistent with the findings of Fei et al. who conclude that “the off-diagonal elements are generally larger for downgrades than upgrades.” ([5], p. 12)

**Table 1:** Percentage of variation of transition probabilities.

	Upgrading	Downgrading
*χ*_*i*_ = 1	$\;\left(1-q_{i}\right)\frac{1-P_{i}}{P_{i}}100$	−(1 − *q*_*i*_)100
*χ*_*i*_ = 0	−(1 − *q*_*i*_)100	$\left(1-q_{i}\right)\frac{P_{i}}{1-P_{i}}100$

Industry-specific probabilities of success is the instrument for diversifying the effect of credit-class specific tendency variables across industries in the coupling schemes described next.

Assume that N debtors initially present in the portfolio have been classified into *S* ≥ 1 industry sectors. In formal terms, the already considered models without diversification correspond to *S* = 1. Set *s*(*n*) for the industry sector of the debtor indexed by *n* initially. Its credit rating *X*_*n*_(*t*) evolves in time according to Eq (1), while the allocation to an industry sector *s*(*n*) remains unchanged. The probability of success *q*_*X*_*n*_(1),*s*(*n*)_ of *δ*_*n*_ depends now upon both the credit class and the industry sector. That is, in order to run a model, one needs a 2^*M*^-dimensional vector $\vec{\pi }$ of probabilities *π*_*j*_ and an *M* × *S* matrix *Q* containing probabilities of success *q*_*i*,*s*_ of Bernoulli random variables in Eq (1). Thus, the total number of unknowns is *M* ⋅ *S* + 2^*M*^.

Accounting for the fact that the effect of favorable or adverse market factors can vary across industry sectors, the instantaneous migration probabilities ${\hat{P}}_{i,j}\left(\cdot \right)$, involving the corresponding *q*_*i*,*s*_, are now industry-specific as well. The respective modification of the formulas given above is straightforward. Remark that for achieving the same effect within MMC models the Markovian matrices corresponding to expansions and contractions have to be industry-specific.

Along with the two dependence models for *η*_*n*_ already discussed, there is a third one [14]. It assigns the same common component to all migrations characterized by a combination of a credit class and an industry sector. For a given set of parameters, it implies weaker dependence among migrations than in the model 1, but stronger than the model 2 [14]. The three models are summarized in Table 2. If *S* = 1, models 1 and 3 coincide.

**Table 2:** Three coupling schemes.

Model	Reference	Dependence of *η*_*n*_ and *η*_*l*_ (conditioned on tendency vector $\vec{\Pi }$)
1	Kaniovski and Pflug [12]	Identical, if debtors numbered by *n* and *l* belong to the same credit class, otherwise independent
2	Wozabal and Hochreiter [13]	Independent
3	Boreiko et al. [14]	Identical, if the respective debtors belong to the same credit class and the same industry sector, otherwise independent

Letting tendency vectors evolve as a finite time-homogeneous Markov chain, ${\vec{\Pi }}^{t},$ *t* ≥ 1, the next generalization requires substantially more computational recourses for estimating model parameters. Let $P=(p_{i,j})$ be a Markovian matrix with 2^*M*^ rows and 2^*M*^ columns and let

$$\begin{array}{c} P\left\{{\vec{\Pi }}^{t+1}={\vec{\chi }}^{\left(j\right)}|{\vec{\Pi }}^{t}={\vec{\chi }}^{\left(i\right)}\right\}=p_{i,j}. \end{array}$$

That is, P governs the evolution of tendency vectors. The distribution of ${\vec{\Pi }}^{t},\;t\geq 2,$ given that the distribution of ${\vec{\Pi }}^{1}$ is $\vec{\pi }$, will be $\vec{\pi }P^{t-1}$. Here, $P^{t-1}$ stands for the (*t* − 1)-th power of P. In particular,

$$\begin{array}{c} P\left\{{\vec{\Pi }}^{t}={\vec{\chi }}^{\left(j\right)}\right\}={\left[\vec{\pi }P^{t-1}\right]}_{j}, \end{array}$$

where ${\left[\vec{\pi }P^{t-1}\right]}_{j}$ denotes the *j*-th coordinate of the vector $\vec{\pi }P^{t-1}$. If *M* = 1, P corresponds to the regime-switching matrix used in the MMC models. The standard setting without a dynamics is a particular case, when P is the 2^*M*^ × 2^*M*^ identity matrix *I*_2^*M*^_. This is a trivial dynamics: every macroeconomic outcome follows itself with certainty.

A dynamic generalization has been tested for all three specifications of common components already considered. Because the Markovian matrix governing macroeconomic dynamics has to be estimated, the total number of unknown parameters is *M* ⋅ *S* + 2^*M*^ + 2^2*M*^.

The next generalization deals with static credit-class- and industry-specific tendency variables. As a byproduct, industry-specific historic probabilities can be incorporated, a departure from the standard CreditMetrics framework.

While the models specified so far entail at most one means of diversification—industry-specific probabilities *q*_*i*,*s*_, in the models described next up to three such means can be involved: industry-specific historic probabilities, credit-class- and industry-specific tendency variables and *q*_*i*,*s*_.

Let us adapt the notation introduced above to the setting with industry- and credit-class-specific tendency variables. With *M* non-default credit classes and *S* industries, a realization $\vec{\chi }$ of a tendency vector $\vec{\Pi }$ contains *M* ⋅ *S* coordinates. There are 2^*M* ⋅ *S*^ such binary vectors. To refer correctly to the corresponding tendency variables, let us agree that the subsequent blocks of *M* coordinates in $\vec{\chi }$ correspond to the respective industry sectors *s* = 1, 2, …, *S*. In particular, the first *M* coordinates are allocated to the hidden variables characterizing industry sector 1. Numbering these binary vectors and assigning a probability *π*_*i*_ to ${\vec{\chi }}^{\left(i\right)}$ we obtain a distribution $\vec{\pi }$ of a tendency vector. Let coordinate *χ*_*M*(*s* − 1)+*i*_ affect the evolution of debtors from credit class *i* and industry sector *s*. If *χ*_*M*(*s*−1)+*i*_ = 1, all of the random variables *η*_*n*_, such that *X*_*n*_(1) = *i* and *s*(*n*) = *s*, cannot assume the values larger than *i*. If *χ*_*M*(*s*−1)+*i*_ = 0, all of the random variables *η*_*n*_, such that *X*_*n*_(1) = *i* and *s*(*n*) = *s*, take on only the values larger than *i*. Since model 1 requires the same common component for all debtors belonging to a credit class, this parametrization cannot be implemented with industry-specific tendency variables. The remaining two, are considered below.

Assuming that there are *S* industry specific *M* × (*M* + 1) historical migration matrices *P*^(*s*)^ with entries $P_{i,j}^{\left(s\right)}$, the corresponding conditional distribution,

$$\begin{array}{c} P\left\{\eta_{n}=j|\vec{\chi }\right\}=P_{X_{n}\left(1\right),j}^{\left(s(n)\right)}\left(\chi_{M\left(s-1\right)+X_{n}\left(1\right)}\right), \end{array}$$

of *η*_*n*_ reads:

$$\begin{array}{c} P_{i,j}^{\left(s\right)}\left(1\right)=\{\begin{array}{cc} P_{i,j}^{\left(s\right)}/P_{i}^{\left(s\right)} & \;\text{if}\;j\leq i,\\ 0 & \;\text{if}\;j>i; \end{array}\;\text{and}\;\;P_{i,j}^{\left(s\right)}\left(0\right)=\{\begin{array}{cc} P_{i,j}^{\left(s\right)}/\left[1-P_{i}^{\left(s\right)}\right] & \;\text{if}\;j>i,\\ 0 & \;\text{if}\;j\leq i. \end{array} \end{array}$$

Here, $P_{i}^{\left(s\right)}=P_{i,1}^{\left(s\right)}+P_{i,2}^{\left(s\right)}+\ldots +P_{i,i}^{\left(s\right)}$. If $P\left\{\Pi_{M(s-1)+i}=1\right\}=P_{i}^{\left(s\right)}$, this definition implies that each migration in industry sector *s* is governed by *P*^(*s*)^. (It is assumed that $P_{i}^{\left(s\right)}\in \left(0,1\right)$.) To guarantee that $P_{i}^{\left(s\right)}=P\left\{\Pi_{M(s-1)+i}=1\right\}$, the following analytic dependence between distribution $\vec{\pi }$ and Markovian transition matrices *P*^(*s*)^ must hold true:

$$\begin{array}{c} P_{i}^{\left(s\right)}=\sum_{j=1}^{2^{M\times S}}I_{\left\{\chi_{M(s-1)+i}^{\left(j\right)}=1\right\}}\pi_{j}. \end{array}$$ (3)

Its left and right hand sides are different expressions for the probability that macroeconomic factors are favorable for debtors belonging to industry sector *s* and credit class *i*. Here $I_{\left\{A\right\}}$ denotes the indicator of a statement *A*:

$$\begin{array}{c} I_{\left\{A\right\}}=\{\begin{array}{c} 1\;\text{if}\;A\;\text{holds}\;\text{true},\\ 0\;\text{if}\;A\;\text{is}\;\text{false}. \end{array} \end{array}$$

Instantaneous migration probabilities ${\hat{P}}_{i,j}^{\left(s\right)}\left(\cdot \right)$ can be defined in this case as well. The following three factors render them industry-specific: first, macroeconomic conditions represented by *χ*_*M*(*s*−1)+*X*_*n*_(1)_, second, *q*_*i*,*s*_ and, third, $P_{i,j}^{\left(s\right)}$. The total number of unknown parameters is *M* ⋅ *S* + 2^*M*⋅*S*^. Here, a dynamic setting can be considered as well. However, the number of unknowns is huge in this case. In particular, a 2^*M*⋅*S*^ × 2^*M*⋅*S*^ transition matrix P for tendency variables has to be estimated.

## Input data

We used Ratingsdirect database and RatingsXpress dataset in DataStream to download the rating histories of all debtors from 35 OECD countries, who had been rated by the S&P’s agency during the period from 1991 to the end of 2015. Debtors with only NR available were removed. We controlled the lists for excluding potential duplicates. As a result, 13613 debtors coming from 34 countries (Latvia had no borrowers identified) were left. For each of the OECD countries involved, the total number of firms, differentiating them between corporate and financial debtors, is given in Table 3. S1 File contains transition counts for 7 credit classes and 6 industry sectors. The industries are listed in Table 4. Counts corresponding to other combinations of *M* and *S* tested in the paper, can be obtained by summation over the respective credit classes and/or industry sectors.

**Table 3:** OECD countries covered by dataset.

Country	Corporate debtors	Financial debtors	Total
Australia	195	169	364
Austria	17	51	68
Belgium	24	39	63
Canada	462	159	621
Chile	26	11	37
Czech Republic	7	13	20
Denmark	20	31	51
Estonia	1	0	1
Finland	20	30	50
France	161	291	452
Germany	134	767	901
Greece	12	14	26
Hungary	10	18	28
Iceland	1	5	6
Ireland	31	71	102
Israel	7	3	10
Italy	55	153	208
Japan	414	214	628
Korea, Republic of	50	29	79
Luxembourg	34	63	97
Mexico	95	48	143
Netherlands	114	114	228
New Zealand	32	65	97
Norway	20	28	48
Poland	12	19	31
Portugal	12	32	44
Slovakia	3	6	9
Slovenia	0	2	2
Spain	30	71	101
Sweden	61	70	131
Switzerland	35	79	114
Turkey	13	21	34
United Kingdom	370	363	733
United States of America	4442	3644	8086
Total	6920	6693	13613

**Table 4:** Industry sectors and their SIC codes.

s	Description	SIC code
1	Agriculture, mining and construction	0–1999
2	Manufacturing	2000–3999
3	Transportation, technology and utility	4000–4999
4	Trade	5000–5999
5	Finance	6000–6999
6	Services	7000–8999.

With a reduction (merging *CCC*, *CC* and *C* in a single credit class *C*) of the basic S&P’s classification, *M* = 7 credit ratings are termed as *AAA*, *AA*, *A*, *BBB*, *BB*, *B* and *C*. Consequently, there are 7 + 2^7^ = 135 parameters to estimate for a static model without differentiation – an easy task for a standard desktop computer and the MATLAB optimization software. With this choice of credit classes, a transition matrix governing the dynamics of tendency vectors, contains 2^7^ × 2^7^ = 16384 entries. Such a number of parameters was too demanding for our computational resources and available data. Consequently, the dynamic settings were tested for a smaller number of non-default credit classes. If *M* = 2, there are investment-grade and non-investment-grade debtors. The investment-grade debtors are characterized by the S&P’s ratings from *AAA* to *BBB*, while the non-investment-grade ones occupy the ratings from *BB* and downward. With this specification, 2^4^ = 16 elements of P have to be estimated.

Turning to the number of industries involved and considering *S* = 6 industry sectors, the following figures emerge. If *M* = 2 and tendency variables are industry-specific, there are 2 ⋅ 6 + 2^2⋅6^ = 4108 parameters to estimate. If all necessary derivatives are approximated by finite differences, standard MATLAB solvers, the Interior Point (IP) method and the Sequential Quadratic Programming (SQP) method, require, according to our experience, 12 hours for one iteration in this case. Given that depending upon the choice of an initial approximation between 80 and 400 iterations are necessary, this way of running calculations seems to be excessively time consuming. Using analytical expressions for the first and the second partial derivatives, the necessary time can be reduced to a couple of hours.

With such considerations in mind, the static models with credit-class-specific tendency variables were tested for *M* = 2 and *M* = 7. If *S* = 1, there are 6 and 135 parameters to estimate, while for *S* = 6 the corresponding numbers are 16 and 170. The dynamic models were tested for *M* = 2 and both *S* = 1 and *S* = 6. Then there are 22 and 32 unknowns. The models with credit-class- and industry-specific tendency variables were tested for *M* = 2 and *S* = 6.

If *M* = 7, the numbers from 1 to 7 correspond to the S&P’s credit classes *AAA*, *AA*, *A*, *BBB*, *BB*, *B* and *C*. For *M* = 2, investment-grade and non-investment-grade debtors are indexed by 1 and 2.

Interpreting coordinates of a tendency vector as a binary representation of an integer, let us number the binary strings in a descending order of the corresponding integers assigning 1 to the binary vector with all coordinates equal to 1. In particular, if *M* = 2, then four binary vectors appear in the following order:

$$\begin{array}{c} {\vec{\chi }}^{\left(1\right)}=\left(1,1\right),\;{\vec{\chi }}^{\left(2\right)}=\left(1,0\right),\;{\vec{\chi }}^{\left(3\right)}=\left(0,1\right),\;{\vec{\chi }}^{\left(4\right)}=\left(0,0\right). \end{array}$$

Applying the Standard Industry Classification (SIC), a number *s* was assigned to each of the following industry sectors:

The presented next matrices were estimated as the time averages. For *M* = 7 and *M* = 2, matrix *P* equals

$$\begin{array}{c} (\begin{array}{cccccccc} 0.8943 & 0.0985 & 0.0049 & 0.0011 & 0.0000 & 0.0000 & 0.0003 & 0.0011\\ 0.0060 & 0.9051 & 0.0834 & 0.0043 & 0.0002 & 0.0007 & 0.0002 & 0.0001\\ 0.0010 & 0.0345 & 0.9050 & 0.0557 & 0.0019 & 0.0006 & 0.0003 & 0.0010\\ 0.0011 & 0.0044 & 0.0547 & 0.8867 & 0.0449 & 0.0059 & 0.0009 & 0.0014\\ 0.0005 & 0.0031 & 0.0091 & 0.1057 & 0.7955 & 0.0741 & 0.0052 & 0.0068\\ 0.0007 & 0.0012 & 0.0038 & 0.0100 & 0.0909 & 0.8106 & 0.0507 & 0.0321\\ 0.0014 & 0.0000 & 0.0014 & 0.0027 & 0.0198 & 0.1352 & 0.5747 & 0.2648 \end{array}), \end{array}$$

and, respectively,

$$\begin{array}{c} (\begin{array}{ccc} 0.9786 & 0.0204 & 0.0010\\ 0.0690 & 0.9000 & 0.0310 \end{array}). \end{array}$$

For *M* = 2, industry-specific historical matrices *P*^(*s*)^, *s* = 1, 2, …, 6, are as the following:

$$\begin{array}{c} (\begin{array}{ccc} 0.9795 & 0.0201 & 0.0004\\ 0.0271 & 0.9388 & 0.0340 \end{array}),\;\;(\begin{array}{ccc} 0.9783 & 0.0214 & 0.0003\\ 0.0252 & 0.9404 & 0.0343 \end{array}), \end{array}$$

$$\begin{array}{c} (\begin{array}{ccc} 0.9860 & 0.0128 & 0.0012\\ 0.0427 & 0.9043 & 0.0530 \end{array}),\;\;(\begin{array}{ccc} 0.9676 & 0.0320 & 0.0004\\ 0.0246 & 0.9490 & 0.0264 \end{array}), \end{array}$$

$$\begin{array}{c} (\begin{array}{ccc} 0.9774 & 0.0215 & 0.0011\\ 0.1562 & 0.8245 & 0.0193 \end{array}),\;\;(\begin{array}{ccc} 0.9708 & 0.0288 & 0.0004\\ 0.0190 & 0.9513 & 0.0297 \end{array}). \end{array}$$

## Estimates and their interpretation

Similarity of parameters estimated for different models is an indication that each of them captures the same features of credit-rating migrations. For this reason, the following next analysis focuses on comparison of results obtained under different assumptions.

Consider first the static models without differentiation. Let us begin with estimates for $q_{j}^{\left(i\right)}$. If *M* = 2, then

$$\begin{array}{c} Q^{\left(1\right)}=\left(0.9815,0.8757\right)\;\;\text{and}\;Q^{\left(2\right)}=\left(0.9816,0.9485\right), \end{array}$$

while

$$\begin{array}{c} Q^{\left(1\right)}=\left(0.8384,0.9040,0.8003,0.9048,0.8366,0.8990,0.7792\right) \end{array}$$

and

$$\begin{array}{c} Q^{\left(2\right)}=\left(0.8345,0.9003,0.9628,0.9506,0.9527,0.9041,0.7462\right) \end{array}$$

for *M* = 7. The strongest dependence on the common component, as measured by the smallest $q_{2}^{\left(1\right)}$ and $q_{2}^{\left(2\right)}$ or $q_{7}^{\left(1\right)}$ and $q_{7}^{\left(2\right)}$, exhibit migrations of non-investment-grade debtors or the debtors rated by *C*. Since these are the credit classes with the lowest creditworthiness, our results are consistent with the two stylized facts stating that riskier credit classes are affected stronger than more secure ones by macroeconomic conditions and that credit rating migrations in riskier credit classes are stronger dependent. See, for example, [16]. Recall that in our case, *q*_*i*_ scales both the impact of macroeconomic factors on conditional migrations probabilities and the strength of dependence among migrations. For scheme 1, Table 5 presents the percentage of variation of downgrading probabilities in the case of *M* = 7. The quoted values evidence that the downgrading probabilities are affected *much* stronger by adverse macroeconomic conditions than by favorable ones. In particular, the percentage corresponding to adverse macroeconomic conditions exceeds more than 20 times its counterpart for favorable macroeconomic conditions, if the debtors rated by *B* are considered. The value 61.3% estimated for the debtors rated at *C* is consistent with 54.998% obtained by the MMC method for the debtors rated at *CCC*. ([5], p. 13) In this paper a S&P’s dataset covering the period 1981–2006 is used. Recall that our *C* rating combines *C*, *CC* and *CCC* of the S&P’s classification. The amplitude of deviation of the conditional default probabilities from their historical counterparts is illustrated by Table 6. It contains the conditional default probabilities corresponding to the percentages quoted in Table 5 as well as the unconditional default probabilities.

**Table 5:** *M* = 7, model 1: Percentages of variation of a downgrading probability.

*χ*_*i*_\*i*	1	2	3	4	5	6	7
*χ*_*i*_ = 1	−16.2	−9.6	−20.0	−9.5	−16.3	−10.1	−22.1
*χ*_*i*_ = 0	136.7	98.4	315.7	169.8	173.4	211.6	61.3

**Table 6:** *M* = 7, model 1: Conditional vs. historical default probabilities.

*i*	1	2	3	4	5	6	7
${\hat{P}}_{i,8}\left(1\right)$	0.0009	0.0001	0.0008	0.0013	0.0057	0.0289	0.1976
${\hat{P}}_{i,8}\left(0\right)$	0.0026	0.0002	0.0042	0.0038	0.0186	0.0680	0.4271
*P*_*i*,8_	0.0011	0.0001	0.0010	0.0014	0.0068	0.0321	0.2648

Now let us consider the distributions ${\vec{\pi }}^{\left(i\right)}$ estimated for the models without differentiation. If *M* = 2, the following probabilities were obtained:

$$\begin{array}{c} \pi_{1}^{\left(1\right)}=0.9506,\;\pi_{2}^{\left(1\right)}=0.0280,\;\pi_{3}^{1)}=0.0184,\;\pi_{3}^{\left(1\right)}=0.0030; \end{array}$$

$$\begin{array}{c} \pi_{1}^{\left(2\right)}=0.9502,\;\pi_{2}^{\left(2\right)}=0.0285,\;\pi_{3}^{\left(2\right)}=0.0188,\;\pi_{4}^{\left(2\right)}=0.0025. \end{array}$$

These values are nearly identical. The coefficients of correlation *R*_1,2_ between non-deteriorating tendencies governing migrations of investment- and non-investment-grade debtors are very close as well: $R_{1,2}^{\left(1\right)}=0.0916$ and $R_{1,2}^{\left(2\right)}=0.0745$. Consequently, both models reveal the same macroeconomic pattern: factors affecting investment- and non-investment-grade debtors are weakly positively correlated, nearly independent.

For *M* = 7, the distributions ${\vec{\pi }}^{\left(1\right)}$ and ${\vec{\pi }}^{\left(2\right)}$ are given in Appendix. According to model 1 (2), there are 11 (13) realizations of tendency vector whose probabilities exceed 0.005 and one of them occurs with probability 1.0000 (0.9964). (For a larger threshold of 0.01, in both cases there are 11 realizations and the corresponding probabilities are 1.0000 and 0.9831.) Probability of the event that all tendency variables attain the value 1 is $\pi_{1}^{\left(1\right)}=0.5720$ and $\pi_{1}^{\left(2\right)}=0.5661$, correspondingly. Interpreting conceptually these values, we conclude that according to both models approximately 57% of years from the period between 1991 and 2015 were favorable for all debtors rated by the S&P’s. Corresponding to an extreme aggregation of credit classes, the analogous probabilities evaluated for *M* = 2 are unrealistically high.

Set *R*_*i*,*j*_ for the coefficient of correlation between tendency variables Π_*i*_ and Π_*j*_. The values $R_{i,j}^{\left(1\right)}$ and $R_{i,j}^{\left(2\right)}$ for *M* = 7 are given next, below and above the main diagonal:

$$\begin{array}{c} (\begin{array}{ccccccc} 1.0000 & 0.1479 & -0.0115 & -0.0023 & 0.1535 & 0.1603 & -0.0417\\ 0.2076 & 1.0000 & 0.5349 & 0.0114 & 0.6327 & 0.8614 & 0.2455\\ -0.0864 & -0.0786 & 1.0000 & 0.3182 & 0.5452 & 0.5579 & 0.0478\\ -0.0814 & -0.0740 & -0.0595 & 1.0000 & 0.0140 & 0.0170 & -0.0870\\ -0.1055 & -0.0959 & 0.8191 & -0.0727 & 1.0000 & 0.7622 & 0.3453\\ 0.2220 & 0.4877 & 0.0901 & -0.0711 & 0.3890 & 1.0000 & 0.3317\\ -0.0031 & 0.2658 & 0.4189 & -0.1421 & 0.5114 & 0.3522 & 1.0000 \end{array})\text{.} \end{array}$$

The sign of 14 out of 21 off-diagonal entries coincides for both schemes. In other words, in 67% of cases the predicted directions of macroeconomic development (an expansion or a recession) are the same. Since some of the correlations are small in absolute value, the impact of eventual calculation errors may not be excluded.

To avoid potential misinterpretation discussing correlations, note that, while there is no relation between the number of positively correlated tendency variables and values of the corresponding coefficients of correlation, the number of negatively correlated tendency variables and the smallest value of correlations between them are related. (Remark that the absolute value of the smallest coefficient of correlation is the largest.) In order to grasp an intuition behind this phenomenon, consider *m* ≥ 2 exchangeable random variables *ζ*_*i*_, each taking values 0 and 1. Let $C\text{orr}(\zeta_{i},\zeta_{j})=c$. Since $V\;\text{ar}(\zeta_{1}+\zeta_{2}+\ldots +\zeta_{m})\geq 0$, it follows that $c\geq -\frac{1}{m-1}$.

Let us turn to the first generalization of the basic setting—the static models with industry- and credit-class-specific *q*_*i*,*s*_ as a (single) means of differentiation. Let us begin with the *Q*^(*i*)^ estimated for *M* = 7:

$$\begin{array}{c} (\begin{array}{cccccc} 0.9999 & 0.8715 & 0.9956 & 0.9999 & 0.8072 & 0.9999\\ 0.7053 & 0.9782 & 0.9825 & 0.9999 & 0.8318 & 1.0000\\ 0.9977 & 1.0000 & 1.0000 & 1.0000 & 0.8035 & 0.9918\\ 1.0000 & 1.0000 & 1.0000 & 1.0000 & 0.8991 & 0.9920\\ 0.9463 & 0.9057 & 0.9850 & 0.9076 & 0.7717 & 0.8356\\ 0.9573 & 0.9572 & 0.9152 & 0.9867 & 0.8661 & 0.9819\\ 0.9579 & 0.7027 & 0.6371 & 0.7794 & 0.9065 & 0.8537 \end{array}), \end{array}$$

$$\begin{array}{c} (\begin{array}{cccccc} 1.0000 & 0.8229 & 0.9805 & 1.0000 & 0.8040 & 1.0000\\ 0.6970 & 0.9801 & 0.9824 & 1.0000 & 0.8152 & 0.9983\\ 1.0000 & 1.0000 & 0.9545 & 0.9956 & 0.9408 & 0.9823\\ 1.0000 & 0.8492 & 0.4990 & 1.0000 & 1.0000 & 0.9705\\ 1.0000 & 0.9198 & 0.8059 & 0.9628 & 1.0000 & 0.9722\\ 0.9243 & 0.9034 & 0.7962 & 0.9495 & 0.9398 & 0.9591\\ 0.9850 & 0.7097 & 0.5671 & 0.7056 & 0.9934 & 0.9486 \end{array}), \end{array}$$

$$\begin{array}{c} (\begin{array}{cccccc} 1.0000 & 0.9050 & 1.0000 & 1.0000 & 0.8349 & 0.9564\\ 0.7064 & 0.9828 & 0.9834 & 1.0000 & 0.8318 & 0.9417\\ 1.0000 & 1.0000 & 1.0000 & 1.0000 & 0.8036 & 0.9820\\ 0.5250 & 0.6396 & 0.5408 & 0.7772 & 0.8908 & 0.7288\\ 0.6638 & 0.6718 & 0.8221 & 0.7303 & 0.8186 & 0.6144\\ 0.9216 & 0.9297 & 0.8729 & 0.9483 & 0.8758 & 0.9620\\ 0.7896 & 0.7264 & 0.6727 & 0.6721 & 0.8119 & 0.9729 \end{array}). \end{array}$$

Now it is not always true that the strongest dependence on the common component, as measured by the corresponding entry of *Q*, is observed for the credit class *C*. In fact, $q_{7,3}^{\left(1\right)}=0.6371$, $q_{4,3}^{\left(2\right)}=0.4990$ and $q_{4,1}^{\left(3\right)}=0.5250$ are the smallest values for the respective models. Consequently, dependence among migrations is the strongest in these credit-classes and industries. Differently to what is observed when debtors are not classified into industry sectors, some credit classes and industries are driven exclusively by idiosyncratic factors. The largest number 4 of idiosyncratically (or almost idiosyncratically) migrating credit classes exhibits the sector of trade. This observation holds for models 1 and 2. The same number of independently evolving credit classes are in the sector of services according to model 1 and in the sector of agriculture, mining and construction according to model 2. Moreover, migrations of debtors rated at *A* are independent (or almost independent) in all industry sectors, except for the financial sector, according to model 3. Similarly, migrations of debtors rated at *A* and at *BBB* are independent (or almost independent) in all industry sectors, except for the finance sector, according to model 1. The last conclusion does not contradict to the statement that, for a given set of parameters, this model is characterized by the strongest dependence pattern. In fact, entries of *Q* represent a “reaction” of the corresponding model to the transition counts. Since the same counts are used to test all models, this “reaction” has to be the weakest in the case of model 1.

For *M* = 7, the estimated distributions ${\vec{\pi }}^{\left(i\right)}$ of tendency vectors are quoted in Appendix. Probability of the event that all tendency variables attain the value 1 is as the following: $\pi_{1}^{\left(1\right)}=0.4998$, $\pi_{1}^{\left(2\right)}=0.4992$ and $\pi_{1}^{\left(1\right)}=0.5886$. For models 1 and 2, these values are 14% and, respectively, 13% lower than their analogs reported for *S* = 1. For model 3, the value is close to its counterpart. (Recall that models 1 and 3 coincide if *S* = 1.) Having all tendency variable equal to 1, this macroeconomic scenario is favorable for all debtors in the S&P’s dataset. Let us estimate the probability

$$\begin{array}{c} P_{+,+}^{\left(i\right)}=\sum_{j=1}^{128}\chi_{1}^{\left(j\right)}\cdot \chi_{2}^{\left(j\right)}\cdot \pi_{j}^{\left(i\right)} \end{array}$$

of the macroeconomic outcome favorable for debtors rated at *AAA* and *AA*. Since $P_{+,+}^{\left(1\right)}=0.8293$, $P_{+,+}^{\left(2\right)}=0.8318$ and $P_{+,+}^{\left(3\right)}=0.8329$, we conclude that approximately 83% of years from the period 1991–2015 were favorable for debtors rated at *AAA* and *AA*. Probability

$$\begin{array}{c} P_{-,-}^{\left(i\right)}=\sum_{j=1}^{128}\left(1-\chi_{1}^{\left(j\right)}\right)\cdot \left(1-\chi_{2}^{\left(j\right)}\right)\cdot \pi_{j}^{\left(i\right)} \end{array}$$

of the macroeconomic event adverse for debtors rated at *AAA* and *AA* is as the following: $P_{-,-}^{\left(1\right)}=0.0239$, $P_{-,-}^{\left(2\right)}=0.0264$ and $P_{-,-}^{\left(3\right)}=0.0269$.

Table 7 characterizes the support of ${\vec{\pi }}^{\left(i\right)}$. For a threshold *ϵ*, *N* denotes the number of outcomes whose probabilities exceed *ϵ* and *Pr* stands for the sum of these probabilities. (Conceptually, *Pr* is the probability that at least one of the outcomes occurs.) This pattern of concentration resembles closely its counterpart reported for *S* = 1. Consequently, the distribution of tendency variables seems to be not affected by the diversification mechanism based on industry-specific *q*_*i*,*s*_. This is an intuitively plausible conclusion given that the part of the model defining tendency variables remains unchanged. Correlations *R*^(*i*)^ are given next. The first of the matrices contains $R_{i,j}^{\left(1\right)}$ below the main diagonal and $R_{i,j}^{\left(2\right)}$ above it.

$$\begin{array}{c} (\begin{array}{ccccccc} 1.0000 & 0.1942 & 0.0705 & -0.0814 & 0.0268 & 0.2081 & -0.0118\\ 0.1658 & 1.0000 & 0.3559 & -0.0740 & 0.2704 & 0.4444 & 0.0226\\ -0.0802 & 0.1349 & 1.0000 & 0.2124 & 0.6019 & 0.3419 & 0.1098\\ -0.0774 & 0.0581 & 0.0996 & 1.0000 & -0.0727 & -0.0711 & -0.1421\\ -0.0989 & -0.0886 & -0.0655 & -0.0677 & 1.0000 & 0.2597 & 0.0356\\ 0.1772 & 0.3707 & -0.0712 & -0.0676 & -0.0883 & 1.0000 & 0.3704\\ -0.0291 & 0.0039 & -0.1441 & -0.1390 & 0.5058 & 0.3941 & 1.0000 \end{array}), \end{array}$$

$$\begin{array}{c} (\begin{array}{ccccccc} 1.0000 & 0.2004 & -0.0864 & -0.0814 & -0.1055 & 0.4065 & 0.1122\\ 0.2004 & 1.0000 & 0.2235 & -0.0740 & -0.0959 & 0.4466 & 0.1501\\ -0.0864 & 0.2235 & 1.0000 & -0.0595 & -0.0772 & -0.0756 & -0.1509\\ -0.0814 & -0.0740 & -0.0595 & 1.0000 & 0.7712 & -0.0711 & 0.3944\\ -0.1055 & -0.0959 & -0.0772 & 0.7712 & 1.0000 & -0.0922 & 0.5114\\ 0.4065 & 0.4466 & -0.0756 & -0.0711 & -0.0922 & 1.0000 & 0.5007\\ 0.1122 & 0.1501 & -0.1509 & 0.3944 & 0.5114 & 0.5007 & 1.0000 \end{array}). \end{array}$$

Models 1 and 3 seem to be coherent in indicating the direction of macroeconomic tendencies: 16 out of 21 off-diagonal entries, or 76%, have the same sign. A comparison of models 1 and 2 reveals less coherence: 13 out of 21 off-diagonal entries, or 62%, have the same sign. Finally, these correlations seem to be similar to their counterparts estimated for the models without differentiation: 14 (17) or 67% (81%) of them preserve their sign in the case of models 1 and 2. Again, we see that the distribution of tendency variables seems to be not strongly affected by the diversification mechanism based on industry-specific *q*_*i*,*s*_.

**Table 7:** *M* = 7, *S* = 6, distribution ${\vec{\pi }}^{\left(i\right)}$: Concentration measure.

Threshold *ϵ*	1		2		3	
	*N*	*Pr*	*N*	*Pr*	*N*	*Pr*
0.005	12	0.9978	13	1.0000	12	1.0000
0.01	10	0.9839	13	1.0000	12	1.0000

For the case of 2 non-default credit classes the following matrices *Q*^(*i*)^ were estimated:

$$\begin{array}{c} (\begin{array}{cccccc} 0.9608 & 1.0000 & 0.6542 & 1.0000 & 1.0000 & 1.0000\\ 0.9977 & 0.9433 & 0.9826 & 0.9991 & 0.8024 & 0.9550 \end{array})\text{,} \end{array}$$

$$\begin{array}{c} (\begin{array}{cccccc} 1.0000 & 1.0000 & 1.0000 & 1.0000 & 0.9656 & 1.0000\\ 0.9529 & 0.9402 & 0.8831 & 0.9753 & 0.9759 & 0.9785 \end{array})\text{,} \end{array}$$

$$\begin{array}{c} (\begin{array}{cccccc} 0.9608 & 1.0000 & 0.6542 & 1.0000 & 1.0000 & 1.0000\\ 0.6149 & 0.5956 & 0.9320 & 0.5109 & 0.8110 & 0.4884 \end{array})\text{.} \end{array}$$

Similar to what is observed while analyzing results for *M* = 7, integrating debtors into bigger groups eliminates idiosyncratic migrations. Now, when all debtors are allocated to 6 industry sectors, some entries of *Q* are equal to 1 implying that the respective credit classes and industry sectors are driven exclusively by idiosyncratic forces. Without the allocation, all entries of *Q* are smaller than 1 and, consequently, no idiosyncracy is possible. As measured by the probabilities of success *q*_*i*,*s*_ in Eq (1), there is no similarity of the dependence patterns exhibited by the three coupling schemes. In fact, the first rows of *Q*^(1)^ and *Q*^(3)^ coincide. Consequently, the dependence patterns in the investment-grade credit class are identical according to these models. At the same time, the entries characterizing non-investment-grade debtors are different. If the corresponding *q*_*i*,*s*_ are equal to 1 or they are close to this value, the effect of macroeconomic conditions, as transmitted by the respective common component, is non-existent or weak. Contrary to the general tendency of nearly independent migrations of investment-grade debtors, $q_{1,3}^{\left(1\right)}=q_{1,3}^{\left(3\right)}=0.6542$. Consequently, models 1 and 3 imply very strong dependence in the sector of transportation. For non-investment-grade debtors in the industry sectors 1, 2, 4 and 6, model 3 predicts even stronger dependence.

Let us consider another implication of small $q_{2,s}^{\left(3\right)}$ – high default probabilities under adverse macroeconomic conditions and strong variation of these probabilities with respect to the historical value. Recall that the default probability of a representative agent equals

$$\begin{array}{c} {\hat{P}}_{2,3}^{\left(s\right)}\left(0\right)=q_{2,s}^{\left(3\right)}P_{2,3}+\left(1-q_{2,s}^{\left(3\right)}\right)\frac{P_{2,3}}{1-P_{2,3}} \end{array}$$

and the percentage of its variation with respect to the long-run counterpart is

$$\begin{array}{c} \Delta_{2,3}^{\left(s\right)}\left(0\right)=\left(1-q_{2,s}^{\left(3\right)}\right)\frac{P_{2,3}}{1-P_{2,3}}100. \end{array}$$

These values are given in Tables 8 and 9. Note that, even if the conditional transition probabilities are the same for all industries, the *instantaneous transition probabilities are industry-specific*. This is an effect of credit-class- and industry-specific weights *q*_*i*,*s*_ in Eq (1). They differentiate the impact of the same (credit-class-specific) macroeconomic factors across industries.

**Table 8:** *M* = 2, model 3: ${\hat{P}}_{2,3}^{\left(s\right)}\left(0\right)$.

*s*	1	2	3	4	5	6
	0.4042	0.4229	0.0969	0.5049	0.2141	0.5267

**Table 9:** *M* = 2, model 3: $\Delta_{2,3}^{\left(s\right)}\left(0\right)$.

*s*	1	2	3	4	5	6
	1203.7	1264.1	212.6	1528.8	590.8	1599.2

Since the maximum likelihood estimates of *π*_*j*_ coincide for models 1 and 2, only the values for models 2 and 3 are quoted next:

$$\begin{array}{c} \pi_{1}^{\left(2\right)}=0.9476,\;\pi_{2}^{82)}=0.0310,\;\pi_{3}^{82)}=0.0214; \end{array}$$

$$\begin{array}{c} \pi_{1}^{\left(3\right)}=0.9690,\;\pi_{2}^{83)}=0.0096,\;\pi_{4}^{\left(3\right)}=0.0214. \end{array}$$

Coefficients of correlation between non-deteriorating tendencies shaping migrations of investment- and non-investment-grade debtors are: $R_{1,2}^{\left(2\right)}=-0.0264$ and $R_{1,2}^{\left(3\right)}=0.8259$. Consequently, macroeconomic factors affecting investment-grade and non-investment-grade debtors seem to be weakly correlated, nearly independent, for models 1 and 2. Since the coefficient is negative, there can be a (slight) substitution effect. In other words, investment-grade and non-investment-grade debtors compete for the market place. according to model 3, the macroeconomic factors appear to be almost identical in their direction as implied by the coefficient of correlation close to 1. None of the distributions reported here matches its counterpart estimated for *S* = 1.

Consider the dynamic models. If *M* = 2 and *S* = 6, distributions of tendency variables $\vec{\pi }$ and transitions matrices P coincide for all three coupling schemes:

$$\begin{array}{c} \pi_{1}=0.9484,\;\pi_{2}=0.0302,\;\pi_{3}=0.0206,\;\pi_{4}=0.0008; \end{array}$$

$$\begin{array}{c} (\begin{array}{cccc} 0.9803 & 0.0013 & 0.0184 & 0.0000\\ 0.0002 & 0.8716 & 0.1279 & 0.0003\\ 0.8722 & 0.1276 & 0.0001 & 0.0001\\ 0.0006 & 0.8893 & 0.0011 & 0.1089 \end{array}). \end{array}$$

For *S* = 1, the distributions $\vec{\pi }$ and the Markovian matrices are as the following:

$$\begin{array}{c} \pi_{1}^{\left(1\right)}=0.9505,\;\pi_{2}^{\left(1\right)}=0.0281,\;\pi_{3}^{\left(1\right)}=0.0185,\;\pi_{4}^{\left(1\right)}=0.0029; \end{array}$$

$$\begin{array}{c} \pi_{1}^{\left(2\right)}=0.9501,\;\pi_{2}^{\left(2\right)}=0.0285,\;\pi_{3}^{\left(2\right)}=0.0189,\;\pi_{4}^{\left(2\right)}=0.0025; \end{array}$$

$$\begin{array}{c} P^{\left(1\right)}=(\begin{array}{cccc} 0.9825 & 0.0042 & 0.0132 & 0.0001\\ 0.0001 & 0.7650 & 0.2011 & 0.0338\\ 0.8971 & 0.0326 & 0.0001 & 0.0702\\ 0.0078 & 0.6983 & 0.0965 & 0.1974 \end{array}), \end{array}$$

$$\begin{array}{c} P^{\left(2\right)}=(\begin{array}{cccc} 0.9687 & 0.0113 & 0.0187 & 0.0014\\ 0.4061 & 0.5456 & 0.0285 & 0.0198\\ 0.9470 & 0.0190 & 0.0141 & 0.0199\\ 0.1205 & 0.7423 & 0.0249 & 0.1123 \end{array}). \end{array}$$

Since $\vec{\pi }P=\vec{\pi }$ in all cases, the *hidden Markov chain is stationary* and $\vec{\pi }$ is its *steady-state distribution*.

In the case of *S* = 6, macroeconomic conditions favorable for all debtors cannot be followed by adverse conditions for them. In fact, *p*_1,4_ = 0. Also *p*_1,3_ is more than ten times greater than *p*_1,2_. Consequently, after a favorable period, a worsening scenario is more likely for non-investment-grade debtors than for investment-grade ones. In the same way, after a crisis the most probable is recovery of the investment grade firms and the least probable is a favorable period for all debtors. The respective probabilities are *p*_4,2_ = 0.8893 and *p*_4,1_ = 0.0006. In sum, the dynamics of a business cycle implied by P is plausible. The steady-state distribution $\vec{\pi }$ exhibits a correlation pattern similar to its static counterparts, observed for models 1 and 2. In fact, *R*_1,2_ = −0.0039 implies that the macroeconomic factors shaping migrations of investment- and non-investment-grade debtors are very weakly dependent. Also, a slight competition for the market place is possible.

Consider the parameters estimated for the dynamic model without diversification. That is, *S* = 1 and *M* = 2. The Markovian matrix $P^{\left(1\right)}$ corresponds to essentially the same business cycle dynamics as we have discussed. A more likely complete recovery after any crisis is implied by $P^{\left(2\right)}$. In fact, $p_{i,1}^{\left(2\right)},\;i=1,\;,2,\;3,$ are larger then the corresponding entries of all other Markovian 2 × 2 matrices quoted here. This is again an effect of integrating debtors into bigger groups: the corresponding macroeconomic scenarios merge as well. Distributions ${\vec{\pi }}^{\left(i\right)}$ are very similar for both schemes and they closely resemble their static counterparts.

The values *θ* = 0.972 and *φ* = 0.759 for the diagonal elements of the quarterly regime-switching matrix in [5], imply the following annual regime-switching matrix:

$$\begin{array}{c} (\begin{array}{cc} 0.9267 & 0.0733\\ 0.6405 & 0.3595 \end{array}). \end{array}$$

It is difficult to compare directly this matrix with ours. In fact, our characterization is more detailed. In particular, we distinguish an expansion for the whole synthetic economy from an expansion in the investment-grade credit class accompanied with a recession affecting non-investment grade debtors. Consequently, one transition within the MMC approach corresponds to several scenarios in our case. Note that a generalization with three regimes is considered in [5].

To assess quantitatively the model quality improvement due to introducing the macroeconomic dynamics, let us use the likelihood function of the dynamic model as a yardstick. Note that the identity matrix is a particular Markovian transition matrix for tendency vectors. Consequently, a solution of a static model, combined with the identity 4 × 4 matrix *I*_4_, will be a feasible point of the maximum likelihood estimator of the respective dynamic model. This observation allows to compare solutions of a static and the corresponding dynamic models. To this end, denote by ${\bar{L}}_{i}$ the maximum value of the likelihood function for the dynamic model *i*. Also let ${\tilde{L}}_{i}$ be the value of this function at the point obtained by combining a solution of the corresponding static model and *I*_4_. Table 10 contains ${\bar{L}}_{i}$ and ${\tilde{L}}_{i}$. Since for some models these values differ too much and it is difficult to compare them, Table 11 characterizes the respective variation in the log-scale. For models 1 and 2, the values obtained for *S* = 6 and *S* = 1 are separated by a slash.

**Table 10:** Likelihood values for comparing static and dynamic models.

Model	1	2	3
${\bar{L}}_{i}$	*e*^907.54^/*e*^731.01^	*e*^328.59^/*e*^241.96^	*e*^1049.55^
${\tilde{L}}_{i}$	*e*^778.49^/*e*^726.49^	*e*^328.59^/*e*^241.95^	*e*^921.06^
$\;\frac{{\tilde{L}}_{i}}{{\bar{L}}_{i}}$	*e*^−129.05^/*e*^−4.52^	1.00/*e*^−0.01^	*e*^−128.49^

**Table 11:** Percentage of improvement in log-scale.

Model	1	2	3
$\frac{\ln{\bar{L}}_{i}-\ln{\tilde{L}}_{i}}{\ln{\tilde{L}}_{i}}100$	16.6/0.6	0.0/0.0	14.0

It appears that the dynamic setting does not imply any improvement for model 2, while for the remaining two models there is an impressive improvement. In other words, the trivial stationary Markovian dynamics, where each macroeconomic outcome, as represented by a realization of tendency vector, follows itself with probability one, is not optimal for models 1 and 3. Since the estimated Markovian transition matrices for tendency variables do not coincide with the 4 × 4 identity matrix, *the trivial Markovian dynamics, where each hidden vector follows itself with probability one, is not optimal for all three schemes*. In other word, *dynamic models are more realistic than their static counterparts*.

For *S* = 6 matrices *Q*^(*i*)^ are given next:

$$\begin{array}{c} (\begin{array}{cccccc} 1.0000 & 1.0000 & 1.0000 & 0.9990 & 0.9647 & 1.0000\\ 0.9978 & 0.9433 & 0.9825 & 0.9991 & 0.8024 & 0.9550 \end{array})\text{,} \end{array}$$

$$\begin{array}{c} (\begin{array}{cccccc} 1.0000 & 1.0000 & 1.0000 & 1.0000 & 0.9656 & 1.0000\\ 0.9530 & 0.9403 & 0.8831 & 0.9753 & 0.9758 & 0.9784 \end{array})\text{,} \end{array}$$

$$\begin{array}{c} (\begin{array}{cccccc} 1.0000 & 1.0000 & 1.0000 & 1.0000 & 0.9646 & 1.0000\\ 0.6151 & 0.5956 & 0.9318 & 0.5106 & 0.8110 & 0.4887 \end{array})\text{.} \end{array}$$

It is not surprising that *Q*^(2)^ quoted here is the same as for the static model 2, whereas *Q*^(1)^ and *Q*^(3)^ differ from their counterparts: the dynamics does not imply any improvement for model 2, but it implies huge improvements for the remaining two models. Interestingly enough, both *Q*^(1)^ and *Q*^(3)^ match poorly (well) their counterparts in the case of investment-grade (non-investment-grade) debtors. In particular, the very strong impact of macroeconomic factors on instantaneous transition probabilities of non-investment-grade debtors in industry sectors 1, 2, 4 and 6 observed for the static model 3 maintains. Also, similar to the static settings, the first rows of *Q*^(1)^ and *Q*^(3)^ are practically identical. Consequently, the dynamic models 1 and 3 inherit the pattern of dependence on the common component in the investment-grade credit class from their static counterparts. Contrary to what is observed for *S* = 6, matrices of *Q*^(*i*)^ coincide for the dynamic and the static model *i* in the case of *S* = 1.

Turning to the models with industry- and credit-class-specific tendency variables, the first research question to ask is whether this generalization implies any improvement if compared with the known coupling schemes. To this end, consider the parameters estimated under the assumption of the same for all debtors historical matrix *P*. Let *S* = 6 and *M* = 2.

If macroeconomic tendencies are identical for all industries, the support of ${\vec{\pi }}^{\left(i\right)}$ consists of at most four points, each formed by 6 identical two-dimensional blocks: (1, 1), (1, 0), (0, 1) and (0, 0). Consequently, any element in the support different from these four sample points is an indication that macroeconomic factors do not affect all industries in the same way. In any case, more than 4 sample points in the support would be a sufficient argument in favor of industry-specific tendency variables. Consider the estimated distributions ${\vec{\pi }}^{\left(i\right)}$. Since for both models there are 8 sample points whose probabilities exceed 0.005, it follows that *tendency variables are*, in fact, *industry-specific*.

Table 12 demonstrates that relatively few of the probabilities $\pi_{j}^{\left(i\right)}$ differ from zero. For a threshold *ϵ*, *N* denotes the number of outcomes whose probabilities exceeds *ϵ* and *Pr* stands for the sum of these probabilities. That is, *Pr* is the probability that at least one of the outcomes occurs. Distributions of tendency vectors ${\vec{\pi }}^{\left(i\right)}$ are given in Appendix.

**Table 12:** Common *P*: Concentration measure for ${\vec{\pi }}^{\left(i\right)}$.

Threshold *ϵ*	2		3	
	*N*	*Pr*	*N*	*Pr*
0.005	8	0.9736	8	0.9692
0.01	3	0.9364	3	0.9262

Consider a 6 × 6 matrix *C* containing below (above) the main diagonal coefficients of correlation

$$\begin{array}{c} C_{i,j}=C\text{orr}\left(\Pi_{2(i-1)+1},\Pi_{2(j-1)+1}\right)\left(C\text{orr}\left(\Pi_{2(i-1)+2},\Pi_{2(j-1)+2}\right)\right). \end{array}$$

The value *C*_*i*,*j*_ measures correlation between non-deteriorating tendencies affecting investment-grade (non-investment-grade) debtors from industries *i* and *j*. In the case of identical two-dimensional blocks for each industry sector, all entries of *C*, being correlations of tendency variables with themselves, are equal to one. Consequently, each off-diagonal element of *C* that differs from 1 is an argument in favor of industry-specific hidden variables. (All elements of the main diagonal are equal to 1, because they are $C\text{orr}(\Pi_{2(i-1)+1},\Pi_{2(i-1)+1})$ or $C\text{orr}(\Pi_{2(i-1)+2},\Pi_{2(i-1)+2}$.) The following matrices *C*^(*i*)^ were estimated for models 2 and 3:

$$\begin{array}{c} C^{\left(2\right)}=(\begin{array}{cccccc} \;1.0000 & \;0.2264 & \;0.2264 & \;0.2572 & \;0.2872 & 0.3305\\ \;0.2886 & \;1.0000 & \;0.9371 & \;0.4164 & \;0.3711 & 0.7054\\ \;0.0073 & \;0.0027 & \;1.0000 & \;0.4161 & \;0.3711 & 0.7054\\ -0.0028 & \;0.2114 & \;0.0004 & \;1.0000 & \;0.5714 & 0.3907\\ -0.0084 & -0.0091 & -0.0073 & -0.0094 & \;1.0000 & 0.5988\\ \;0.0054 & \;0.0015 & \;0.0333 & \;0.7618 & -0.0075 & 1.0000 \end{array}), \end{array}$$

$$\begin{array}{c} C^{\left(3\right)}=(\begin{array}{cccccc} \;1.0000 & \;0.5503 & \;0.2821 & \;0.7213 & \;0.5490 & 0.7213\\ \;0.3779 & \;1.0000 & \;0.2898 & \;0.7135 & \;0.8309 & 0.7135\\ \;0.3779 & \;0.3779 & \;1.0000 & \;0.0362 & \;0.2897 & 0.0362\\ -0.0219 & \;0.1893 & -0.0219 & \;1.0000 & \;0.7135 & 1.0000\\ -0.0219 & -0.0219 & -0.0219 & -0.0219 & \;1.0000 & 0.7135\\ -0.0218 & -0.0219 & -0.0218 & \;0.7889 & -0.0219 & 1.0000 \end{array}). \end{array}$$

Since none (only one) of the off-diagonal entries equals 1 in the case of model 2 (3), it follows, once again, that *tendency variables are industry-specific*. Interestingly enough, the unobserved macroeconomic factors affecting investment-grade debtors in the financial sector appear to be independent of what happens in this credit class in the remaining industries. Since the correlations are negative, there seems to be competition for the market place with other investment-grade debtors. This observation holds for both coupling schemes.

The matrix *IN* allows for an additional insight into the structure of dependence between hidden variables or, conceptually, into propagation of a business cycle through the respective industries and credit classes. It is formed by coefficients of correlation, $C\text{orr}(\Pi_{2(l-1)+1},\Pi_{2(s-1)+2})$, between non-deteriorating tendencies affecting an investment-grade debtor from industry *l* and a non-investment-grade one from industry *s*.

$$\begin{array}{c} IN^{\left(2\right)}=(\begin{array}{cccccc} \;0.8031 & \;0.2393 & \;0.2393 & -0.0027 & -0.0024 & \;0.2395\\ \;0.2358 & \;0.8067 & \;0.8067 & \;0.1737 & \;0.3846 & \;0.8067\\ \;0.3613 & \;0.0674 & \;0.0673 & \;0.7018 & \;0.7706 & \;0.3407\\ -0.0082 & \;0.4573 & \;0.4573 & \;0.4574 & -0.0080 & \;0.1719\\ -0.0144 & -0.0144 & -0.0144 & -0.0143 & -0.0143 & -0.0143\\ \;0.0139 & \;0.2987 & \;0.2987 & \;0.2998 & \;0.0175 & \;0.0140 \end{array}), \end{array}$$

$$\begin{array}{c} IN^{\left(3\right)}=(\begin{array}{cccccc} \;0.3074 & -0.0264 & \;0.3074 & -0.0264 & -0.0264 & -0.0264\\ \;0.3073 & \;0.3167 & \;0.8268 & -0.0264 & \;0.3167 & -0.0264\\ \;0.6642 & \;0.3347 & \;0.3479 & \;0.4930 & \;0.3335 & \;0.4930\\ -0.0264 & -0.0264 & \;0.4509 & -0.0264 & -0.0264 & -0.0264\\ -0.0264 & -0.0264 & -0.0264 & -0.0264 & -0.0264 & -0.0264\\ 0.0264 & -0.0264 & \;0.2747 & -0.0264 & -0.0264 & -0.0264 \end{array}). \end{array}$$

The diagonal elements here are analogs of the correlations *R*_1,2_ evaluated for the models involving credit-class specific tendency variables. Consequently, we can compare dependence patterns exhibited by the simpler models with their counterparts corresponding to the models involving credit-class- and industry-specific-tendency variables. In particular, according to the model 3, industry sectors 4, 5 and 6 are characterized by very weak negative correlation between macroeconomic factors affecting the investment-grade and the non-investment-grade debtors. Approximately the same correlation coefficient was estimated for the model 2 with credit-class-specific tendency variables. Moreover, according to the model 2, in the industries 1 and 2, the dependence is almost as strong as it was estimated for the model 3 with credit-class-specific tendency variables.

Correlations in *IN* characterizing the investment-grade debtors of the financial sector, imply almost independence and weak competition for the market place with all non-investment-grade debtors. Summarizing this observation with a similar observation based on the correlations presented in *C*, we conclude that the *macroeconomic factors affecting the investment-grade part of financial sector appear to be not related to the macroeconomic forces driving the rest of the (synthetic) economy*.

In order to assess quantitatively the improvement of model quality due to industry-specific tendency variables let us use the likelihood function of the model with credit-class- and -industry-sector-specific tendency variables as a yardstick.

Recall that a feasible point of an estimator of a model involving credit-class-specific tendency variables has 16 components: 12 of them are entries of *Q* and 4 are probabilities *π*_*i*_. We call it a feasible point of a simpler model in what follows next. A feasible point corresponding to a model with industry- and credit-class-specific tendency variables contains 4108 components: 12 entries of *Q* and 4096 coordinates of $\vec{\pi }$. This is referred to as a feasible point of a complete model. The binary vectors

$$\begin{array}{c} {\vec{\chi }}^{\left(1\right)}=\left(1,1,1,1,1,1,1,1,1,1,1,1\right),\;{\vec{\chi }}^{\left(1366\right)}=\left(1,0,1,0,1,0,1,0,1,0,1,0\right),\; \end{array}$$

$$\begin{array}{c} {\vec{\chi }}^{\left(2719\right)}=\left(0,1,0,1,0,1,0,1,0,1,0,1\right),\;{\vec{\chi }}^{\left(4096\right)}=\left(0,0,0,0,0,0,0,0,0,0,0,0\right),\; \end{array}$$

represent the block structures formed by 11, 10, 01 and 00. In terms of a complete model, each of the block outcomes corresponds to the macroeconomic factors that are not differentiated across industries. For example, ${\vec{\chi }}^{\left(1366\right)}$ implies the macroeconomic conditions favorable for investment-grade debtors in all industries and adverse macroeconomic conditions for non-investment grade debtors in all industries. To avoid bulky notation, let us skip the index *i* in the argument following next. Given a solution, *Q*^#^ and ${\vec{\pi }}^{\#}$, of a simpler model, we define a feasible point, *Q*^(0)^ and ${\vec{\pi }}^{\left(0\right)}$, of the corresponding complete model by setting *Q*^(0)^ = *Q*^#^ and $\pi_{1}^{\left(0\right)}=\pi_{1}^{\#}$, $\pi_{1366}^{\left(0\right)}=\pi_{2}^{\#}$, $\pi_{2719}^{\left(0\right)}=\pi_{3}^{\#}$, $\pi_{4096}^{\left(0\right)}=\pi_{4}^{\#}$. In Table 13, $L_{i}^{*}$ denotes the maximum value of the likelihood function for the complete model *i*. $L_{i}^{\left(0\right)}$ stands for the value of this function at the feasible point obtained with the solution of the respective simpler model. The values presented in Table 13 imply that *a substantial improvement of the model quality, as measured by the corresponding likelihood function, can be achieved by introducing industry-specific hidden variables*. Then it is quite natural, that

$$\begin{array}{c} Q^{\left(2\right)}=(\begin{array}{cccccc} 0.9680 & 0.9798 & 0.6530 & 0.9615 & 0.9656 & 0.9310\\ 0.9023 & 0.9402 & 0.8831 & 0.9601 & 0.7019 & 0.9690 \end{array}) \end{array}$$

and

$$\begin{array}{c} Q^{\left(3\right)}=(\begin{array}{cccccc} 0.9713 & 0.9803 & 0.9566 & 0.9607 & 0.9646 & 0.9304\\ 0.7726 & 0.7185 & 0.8737 & 0.5110 & 0.8111 & 0.4885 \end{array})\text{,} \end{array}$$

do not resemble closely their counterparts evaluated for the simpler models. However, some of the features mentioned earlier persist. In particular, the strong dependence among migrations of non-investment-grade debtors in the industries 4 and 6 observed earlier for model 3. A new phenomenon is that none of the entries equals 1. Consequently, *all migrations are dependent and the instantaneous transition probabilities of all debtors are adjusted according to macroeconomic conditions*. There can be a technical explanation for this phenomenon – accumulation of small errors, due to rounding. Indeed, there are 4108 unknowns in the case of industry-specific hidden variables. Even if the error in estimating each of these values is vanishingly small, due to non-linearity of the likelihood function, the total impact of the errors could push probabilities *q*_*i*,*s*_ away from 1.

**Table 13:** Efficiency’s gain due to modeling industry-specific tendency variables.

Model	2	3
$L_{i}^{*}$	exp(424.44)	exp(1258.15)
$L_{i}^{\left(0\right)}$	exp(194.35)	exp(921.06)
$\frac{L_{i}^{\left(0\right)}}{L_{i}^{*}}$	exp(−230.09)	exp(−337.09)
$\frac{\ln L_{i}^{*}-\ln L_{i}^{\left(0\right)}}{\ln L_{i}^{\left(0\right)}}100$	118	36

In order to test this guess, an additional constraint was introduced in the estimator so that to keep the block structure of tendency variables. Involving 4108 unknowns, the corresponding estimators should be conceptually equivalent to their simpler counterparts with 16 unknowns. For this modification, the following matrices *Q*^(*i*)^ were estimated:

$$\begin{array}{c} Q^{\left(2\right)}=(\begin{array}{cccccc} 0.9722 & 0.9799 & 0.6530 & 0.9636 & 0.9585 & 0.9205\\ 0.9107 & 0.9402 & 0.8831 & 0.9685 & 0.7019 & 0.9690 \end{array})\text{;} \end{array}$$

$$\begin{array}{c} Q^{\left(3\right)}=(\begin{array}{cccccc} 0.9974 & 0.9953 & 0.9952 & 0.9822 & 0.9646 & 0.9783\\ 0.7726 & 0.7185 & 0.8736 & 0.5110 & 0.8111 & 0.4885 \end{array}). \end{array}$$

Distributions of tendency vectors, *π*_1_ = 0.9476, *π*_1366_ = 0.0310, *π*_2731_ = 0.0214 and *π*_*j*_ = 0 for all other *j*, turned out to be identical for both coupling schemes.

While entries of *Q* are very close to their counterparts estimated without the additional requirement for the block structure, they do not match well the values reported for the models, where macroeconomic factors are not differentiated with industry-specific tendency variables. In fact, there is no probability of success *q*_*i*,*s*_ equal to 1. Moreover, the values $q_{1,3}^{\left(2\right)}$ and $q_{1,6}^{\left(2\right)}$ differ from unity so substantially, that the error accumulation argument cannot explain such a difference. At the same time, all $q_{1,s}^{\left(3\right)}$, except for $q_{1,3}^{\left(3\right)}$, are consistent with this argument. Comparing distributions of tendency vectors, we see that the distribution quoted here for model 3 does not correspond to its counterpart. In sum, even if the results quoted here were meant to be conceptually equivalent to what was reported before, they look rather like the estimates for the case of industry-specific tendency variables without the block structure. This informal observation can be supported by an analytic argument. In Table 14, $L_{i}^{*}$ denotes the maximum value of the likelihood function for model *i* with industry-specific tendency variables. The maximum value of this function when the support of the distribution of a tendency vector is additionally restricted to the four block outcomes is denoted by $L_{i}^{\left(0\right)}$. For the data in hand, these values seem to characterize the actual impact due to industry-specific tendency variables. Note that the rounding effect applies now to both estimators.

**Table 14:** Industry-specific tendency variables: Actual effect of block structure.

Model	2	3
$L_{i}^{*}$	exp(424.44)	exp(1258.15)
$L_{i}^{\left(0\right)}$	exp(291.83)	exp(1156.11)
$\frac{L_{i}^{\left(0\right)}}{L_{i}^{*}}$	exp(−132.61)	exp(−102.04)
$\frac{\ln L_{i}^{*}-\ln L_{i}^{\left(0\right)}}{\ln L_{i}^{\left(0\right)}}100$	45	9

Let us turn to the case when each industry sector *s* is governed by its specific historical migration matrix *P*^(*s*)^. In this case, matrices *C* and *IN* are as the following:

$$\begin{array}{c} C^{\left(2\right)}=(\begin{array}{cccccc} \;1.0000 & \;0.1626 & 0.4396 & \;0.1460 & 0.2682 & 0.2076\\ \;0.2798 & \;1.0000 & 0.7808 & \;0.6183 & \;0.3582 & 0.6591\\ \;0.3658 & \;0.3507 & 1.0000 & \;0.6714 & \;0.5644 & 0.5625\\ \;-0.0069 & \;0.2225 & 0.0078 & \;1.0000 & \;0.1631 & 0.2498\\ -0.0081 & -0.0097 & 0.0001 & -0.0166 & \;1.0000 & 0.4238\\ -0.0004 & -0.0046 & 0.0454 & \;0.8330 & -0.0137 & 1.0000 \end{array}), \end{array}$$

$$\begin{array}{c} C^{\left(3\right)}=(\begin{array}{cccccc} 1.0000 & 0.1508 & 0.4192 & \;0.1100 & \;0.2575 & 0.2614\\ 0.2402 & 1.0000 & 0.7641 & \;0.6648 & \;0.3509 & 0.6811\\ 0.3206 & 0.3012 & 1.0000 & \;0.6523 & \;0.5423 & 0.6713\\ 0.0180 & 0.2971 & 0.0413 & \;1.0000 & \;0.1466 & 0.3899\\ 0.0055 & 0.0022 & 0.0164 & -0.0041 & \;1.0000 & 0.4846\\ 0.0121 & 0.0056 & 0.3675 & \;0.6869 & -0.0037 & 1.0000 \end{array}), \end{array}$$

$$\begin{array}{c} IN^{\left(2\right)}=(\begin{array}{cccccc} \;0.7467 & \;0.2176 & \;0.1677 & 0.0025 & 0.0102 & \;0.2397\\ \;0.2106 & \;0.7679 & \;0.6141 & \;0.2547 & \;0.4479 & \;0.8299\\ \;0.5042 & \;0.2834 & \;0.4703 & 0.1703 & \;0.2620 & \;0.3478\\ 0.0151 & \;0.5465 & \;0.4317 & \;0.6324 & -0.0003 & \;0.1879\\ -0.0168 & -0.0173 & -0.0262 & -0.0116 & -0.0058 & -0.0140\\ \;0.0106 & \;0.3896 & \;0.3287 & \;0.4701 & \;0.0316 & \;0.0102 \end{array}), \end{array}$$

$$\begin{array}{c} IN^{\left(3\right)}=(\begin{array}{cccccc} \;0.7261 & \;0.1925 & \;0.1478 & 0.0257 & 0.0372 & \;0.2128\\ \;0.1808 & \;0.7536 & \;0.6025 & 0.3342 & 0.4139 & \;0.8142\\ \;0.2564 & \;0.2506 & \;0.2011 & 0.0514 & 0.0659 & \;0.2770\\ \;0.0264 & \;0.5936 & \;0.4895 & 0.6986 & 0.0535 & \;0.3024\\ -0.0054 & -0.0061 & -0.0169 & 0.0012 & 0.0092 & -0.0018\\ \;0.0018 & \;0.3505 & \;0.2730 & 0.4088 & 0.0211 & \;0.0065 \end{array}). \end{array}$$

Similar to what is observed in the case of the same *P* for all debtors, the macroeconomic factors affecting investment-grade debtors in the financial sector depend very weakly on the macroeconomic factors driving the rest of the synthetic economy and, often, a slight substitution effect can be expected.

Comparing values presented in Tables 12 and 15, we conclude that the distribution $\vec{\pi }$ is slightly more concentrated in the case of industry-specific transition matrices. In fact, for each of the reported thresholds, the number of the corresponding outcomes is larger or not smaller and the probability that at least one of them occurs is higher. The estimated distributions of tendency vectors ${\vec{\pi }}^{\left(2\right)}$ and ${\vec{\pi }}^{\left(3\right)}$ are given in Appendix.

**Table 15:** Industry-specific *P*^(*s*)^: Concentration measure for ${\vec{\pi }}^{\left(i\right)}$.

Threshold *ϵ*	2		3	
	*N*	*Pr*	*N*	*Pr*
0.005	9	0.9766	8	0.9688
0.01	5	0.9501	4	0.9372

Probabilities of success *q*_*i*,*s*_

$$\begin{array}{c} Q^{\left(2\right)}=(\begin{array}{cccccc} 0.9675 & 0.9800 & 0.9453 & 0.9691 & 0.9665 & 0.9320\\ 0.9025 & 0.9425 & 0.8931 & 0.9578 & 0.8532 & 0.9683 \end{array})\text{,} \end{array}$$

$$\begin{array}{c} Q^{\left(3\right)}=(\begin{array}{cccccc} 0.9672 & 0.9812 & 0.9635 & 0.9685 & 0.9654 & 0.9351\\ 0.8993 & 0.9398 & 0.8949 & 0.9585 & 0.7931 & 0.9592 \end{array})\text{.} \end{array}$$

differ from their counterparts evaluated for a single historical matrix *P*. As measured by greater values *q*_*i*,*s*_ (11 out 12 for model 2 and 10 out of 12 for model 3), *migrations are less dependent and the adverse market conditions affect less downgrading probabilities in the case of industry-specific transition matrices*. This fact implies, in particular, that banks using a model based on industry-specific historical matrices would have more capital for lending during downturns than banks using for their risk estimates a single historical migration matrix. In other words, incorporating industry-specific historical matrices in credit risk models improves their predictions.

## Conclusions

This paper proposes a set of probabilistic models for quantifying the impact of macroeconomic conditions on credit rating migrations. Analyzing dynamics of a business cycle, unobserved factors are taken into consideration. The only input required is historical credit-rating migrations. The toolkit is tested on S&P’s data covering the period between 1991 and 2015.

The models are either static or a Markovian macroeconomic dynamics is involved. Each of them entails a coupling scheme. All known coupling schemes are used. In the simplest case, macroeconomic factors are modeled as credit-class-specific. Credit-class- and industry-specific macroeconomic factors are considered in a more detailed analysis. An intermediate approach threats macroeconomic factors as credit-class-specific and involves a micro-founded mechanism diversifying their impact across industries. Subsequent generalizations are quantitatively compared. As a measure of goodness of fit the likelihood function corresponding to the more general model is used. Since in some cases the improvement is huge, we have to use a logarithmic scale for the comparison.

The suggested models alow to estimate conditional migration probabilities as well as probabilities of the corresponding macroeconomic outcomes. There can be a single historical migration matrix for all debtors or industry-specific migration matrices. Variation of the conditional probabilities with respect to their historical counterparts exceeds 1500% in some industries and credit classes. A bank, using a risk model based on conditional probabilities, will better absorb shocks caused by financial and economic stress. This is one of the goals of the Basel III Accord, issued in 2011 by the Basel Committee on Banking Supervision (BCBS). Typically, upgrading probabilities depend less upon macroeconomic conditions than downgrading probabilities. Also downgrading probabilities, defaults in particular, depend much stronger on adverse macroeconomic conditions than on favorable ones. Consequently, our finding concerning conditional probabilities are more illuminating for analyzing downturns than for growth phases. Fortunately, growth phases prevail in the last 25 years. According to our models, approximately 57% of years from period between 1991 and 2015 were favorable for all debtors rated by the S&P’s. For a smaller pool, this value is higher: there were 83% of favorable years for the debtors rated at *AAA* and *AA* by the S&P’s.

All estimated macroeconomic dynamics are stationary. In the majority of cases, a dynamic model overperforms greatly its static counterpart: the corresponding values of goodness of fit allow a meaningful comparison only in a logarithmic scale.

A range of correlations characterizing the interaction of macroeconomic factors driving different credit classes and industries is reported here. One of the most interesting findings is that macroeconomic factors affecting investment-grade debtors in the financial sector are nearly independent of macroeconomic conditions governing all other credit classes and sectors. This observation relies on estimates obtained from four different models and points towards significant differences in the functioning of the financial sector relative to the rest of the economy. We surmise that these differences are partly due to central banks and regulatory authorities pursuing a set of policies that establish a financial safety net—now more so than ever, as the recent global financial and economic crises have increased the need for a prudential policy aimed at managing systemic financial risks.

Here industry sectors are defined by splitting the SIC codes into six subsequent intervals, as it frequently done in empirical analysis. Any other partition in industry sectors of the S&P’s data used in this paper or data of another rating agency could be analyzed in the same way. Moreover, the pool of debtors can be split into groups using different principles. For example, according to market capitalization or other characteristics of their size. If there are enough observations in each of the groups, the estimates can be obtained by the technique presented here.

## Appendix

In all cases logarithm of the respective likelihood function is maximized subject to constraints. The estimates are based on 25 years of observation, from *t* = 1 to *T* = 25, corresponding to 1991 and 2015. In each of the likelihood functions given next, a multiplier that does not contain unknowns is ignored. As such, it is irrelevant for estimation. The likelihood functions of dynamic models are:

$$\begin{array}{c} L_{1}\left(\vec{\pi },Q,P\right)=\prod_{t=1}^{T}\sum_{i=1}^{2^{M}}{\left[\vec{\pi }P^{t-1}\right]}_{i}\prod_{m_{1}=1}^{M}g\left(t,{\vec{\chi }}^{\left(i\right)},m_{1},Q\right), \end{array}$$

$$\begin{array}{c} L_{2}\left(\vec{\pi },Q,P\right)=\prod_{t=1}^{T}\sum_{i=1}^{2^{M}}{\left[\vec{\pi }P^{t-1}\right]}_{i}\prod_{s=1}^{S}\prod_{m_{1}=1}^{M}\prod_{m_{2}=1}^{M+1}f{\left(s,{\vec{\chi }}^{\left(i\right)},m_{1},m_{2},Q\right)}^{I^{t}\left(s,m_{1},m_{2}\right)}, \end{array}$$

$$\begin{array}{c} L_{3}\left(\vec{\pi },Q,P\right)=\prod_{t=1}^{T}\sum_{i=1}^{2^{M}}{\left[\vec{\pi }P^{t-1}\right]}_{i}\prod_{s=1}^{S}\prod_{m_{1}=1}^{M}v\left(t,s,{\vec{\chi }}^{\left(i\right)},m_{1},Q\right). \end{array}$$

Here,

$$\begin{array}{c} g(t,\vec{\chi },m_{1},Q)= \end{array}$$

$$\begin{array}{c} \sum_{i=1}^{M+1}P_{m_{1},i}\left(\chi_{m_{1}}\right)\prod_{s=1}^{S}{\left(q_{m_{1},s}+\frac{1-q_{m_{1},s}}{P_{m_{1},i}}\right)}^{I^{t}\left(s,m_{1},i\right)}\prod_{m_{2}=1,m_{2}\neq i}^{M+1}q_{m_{1},s}^{I^{t}\left(s,m_{1},m_{2}\right)}; \end{array}$$

$$\begin{array}{c} f\left(s,\vec{\chi },m_{1},m_{2},Q\right)=\{\begin{array}{c} \frac{1-q_{m_{1},s}\left(1-P_{m_{1}}\right)}{P_{m_{1}}},\;\;\;\text{if}\;m_{1}\geq m_{2},\;\chi_{m_{1}}=1,\\ \frac{1-q_{m_{1},s}P_{m_{1}}}{1-P_{m_{1}}},\;\;\;\;\;\text{if}\;m_{1}<m_{2},\;\chi_{m_{1}}=0,\\ q_{m_{1},s},\;\;\;\text{otherwise;}\;\;\; \end{array} \end{array}$$

$$\begin{array}{c} v(t,s,\vec{\chi },m_{1},Q)= \end{array}$$

$$\begin{array}{c} \sum_{m_{2}=1}^{M+1}P_{m_{1},m_{2}}\left(\chi_{m_{1}}\right){\left(q_{m_{1},s}+\frac{1-q_{m_{1},s}}{P_{m_{1},m_{2}}}\right)}^{I^{t}\left(s,m_{1},m_{2}\right)}\prod_{j=1,\;j\neq m_{2}}^{M+1}q_{m_{1},s}^{I^{t}\left(s,m_{1},j\right)}; \end{array}$$

*I*^*t*^(*s*, *l*, *k*) denotes the number of debtors in industry sector *s* that moved from credit class *l* to credit class *k* in period *t*.

The following constraints have to be satisfied:

$$\begin{array}{c} \sum_{j=1}^{2^{M}}\pi_{j}=1, \end{array}$$

$$\begin{array}{c} \sum_{j=1}^{2^{M}}{\left[\vec{\pi }P^{t-1}\right]}_{j}I_{\left\{\chi_{i}^{\left(j\right)}=1\right\}}=P_{i},\;i=1,2,\ldots ,M,\;t=1,2,\ldots ,T, \end{array}$$

$$\begin{array}{c} \sum_{j=1}^{2^{M}}p_{i,j}=1,\;i=1,2,\ldots ,2^{M}, \end{array}$$

$$\begin{array}{c} {\left[\vec{\pi }P^{t-1}\right]}_{i}\leq D\pi_{i},\;i=1,2,\ldots ,2^{M},\;t=2,3,\ldots ,T. \end{array}$$

Here, *D* denotes a positive constant. For the numerical results presented in the paper *D* = 2500. Elements of *Q*, P and $\vec{\pi }$ belong to [0, 1].

For likelihood functions of the static models see [13], [14] and [17].

Some of the distributions of tendency vectors mentioned in the text are given next. In all cases, only probabilities exceeding 0.005 are quoted. (If the corresponding outcomes did not form a sure event, the probabilities were normalized.)

**Credit-class specific tendency variables.** *S* = 1 and *M* = 7: $\pi_{1}^{\left(1\right)}=0.5720$, $\pi_{2}^{\left(1\right)}=0.1218$, $\pi_{8}^{\left(1\right)}=0.0266$, $\pi_{9}^{\left(1\right)}=0.0531$, $\pi_{22}^{\left(1\right)}=0.0489$, $\pi_{24}^{\left(1\right)}=0.0106$, $\pi_{33}^{\left(1\right)}=0.0139$, $\pi_{34}^{\left(1\right)}=0.0294$, $\pi_{35}^{\left(1\right)}=0.0181$, $\pi_{65}^{\left(1\right)}=0.0781$, $\pi_{100}^{\left(1\right)}=0.0276$; $\pi_{1}^{\left(2\right)}=0.5681$, $\pi_{2}^{\left(2\right)}=0.1832$, $\pi_{6}^{\left(2\right)}=0.0201$, $\pi_{8}^{\left(2\right)}=0.0079$, $\pi_{9}^{\left(2\right)}=0.0332$, $\pi_{25}^{\left(2\right)}=0.0146$, $\pi_{33}^{\left(2\right)}=0.0140$, $\pi_{36}^{\left(2\right)}=0.0168$, $\pi_{55}^{\left(2\right)}=0.0206$, $\pi_{56}^{\left(2\right)}=0.0153$, $\pi_{65}^{\left(2\right)}=0.08336$, $\pi_{104}^{\left(2\right)}=0.0169$, $\pi_{128}^{\left(2\right)}=0.0055$. *S* = 6 and *M* = 7: $\pi_{1}^{\left(1\right)}=0.5009$, $\pi_{2}^{\left(1\right)}=0.1096$, $\pi_{4}^{\left(1\right)}=0.0462$, $\pi_{6}^{\left(1\right)}=0.0848$, $\pi_{9}^{\left(1\right)}=0.0444$, $\pi_{17}^{\left(1\right)}=0.0445$, $\pi_{33}^{\left(1\right)}=0.0380$, $\pi_{35}^{\left(1\right)}=0.0127$, $\pi_{49}^{\left(1\right)}=0.0058$, $\pi_{57}^{\left(1\right)}=0.0082$, $\pi_{65}^{\left(1\right)}=0.0814$, $\pi_{100}^{\left(1\right)}=0.0236$; $\pi_{1}^{\left(2\right)}=0.4992$, $\pi_{2}^{\left(2\right)}=0.1979$, $\pi_{4}^{\left(2\right)}=0.0248$, $\pi_{5}^{\left(2\right)}=0.0411$, $\pi_{9}^{\left(2\right)}=0.0386$, $\pi_{24}^{\left(2\right)}=0.0158$, $\pi_{25}^{\left(2\right)}=0.0144$, $\pi_{33}^{\left(2\right)}=0.0289$, $\pi_{33}^{\left(2\right)}=0.0309$, $\pi_{35}^{\left(2\right)}=0.0158$, $\pi_{53}^{\left(2\right)}=0.0178$, $\pi_{65}^{\left(2\right)}=0.0793$, $\pi_{100}^{\left(2\right)}=0.0150$, $\pi_{120}^{\left(2\right)}=0.0114$; $\pi_{1}^{\left(3\right)}=0.5886$, $\pi_{2}^{\left(3\right)}=0.0959$, $\pi_{4}^{\left(3\right)}=0.0241$, $\pi_{6}^{\left(3\right)}=0.0330$, $\pi_{14}^{\left(3\right)}=0.0531$, $\pi_{17}^{\left(3\right)}=0.0377$, $\pi_{33}^{\left(3\right)}=0.0246$, $\pi_{36}^{\left(3\right)}=0.0155$, $\pi_{49}^{\left(3\right)}=0.0218$, $\pi_{65}^{\left(3\right)}=0.0624$, $\pi_{68}^{\left(3\right)}=0.0162$, $\pi_{100}^{\left(3\right)}=0.0270$.

**Credit-class- and industry-specific tendency variables.** Common for all industries transition matrix: $\pi_{1}^{\left(2\right)}=0.9457$, $\pi_{9}^{\left(2\right)}=0.0273$, $\pi_{773}^{\left(2\right)}=0.0066$, $\pi_{887}^{\left(2\right)}=0.0068$, $\pi_{961}^{\left(2\right)}=0.0068$, $\pi_{3954}^{\left(2\right)}=0.0068$; $\pi_{1}^{\left(3\right)}=0.9201$, $\pi_{9}^{\left(3\right)}=0.0221$, $\pi_{35}^{\left(3\right)}=0.0097$, $\pi_{99}^{\left(3\right)}=0.0078$, $\pi_{837}^{\left(3\right)}=0.0089$, $\pi_{1302}^{\left(3\right)}=0.0094$, $\pi_{2049}^{\left(3\right)}=0.0134$, $\pi_{3777}^{\left(3\right)}=0.0086$. Industry-specific Markovian matrices: $\pi_{1}^{\left(2\right)}=0.9108$, $\pi_{2}^{\left(2\right)}=0.0060$, $\pi_{9}^{\left(2\right)}=0.0225$, $\pi_{35}^{\left(2\right)}=0.0133$, $\pi_{371}^{\left(2\right)}=0.0125$, $\pi_{838}^{\left(2\right)}=0.0091$, $\pi_{882}^{\left(2\right)}=0.0061$, $\pi_{3073}^{\left(2\right)}=0.0138$, $\pi_{4034}^{\left(2\right)}=0.0059$; $\pi_{1}^{\left(3\right)}=0.9202$, $\pi_{9}^{\left(3\right)}=0.0220$, $\pi_{35}^{\left(3\right)}=0.0099$, $\pi_{131}^{\left(3\right)}=0.0069$, $\pi_{371}^{\left(3\right)}=0.0111$, $\pi_{838}^{\left(3\right)}=0.0081$, $\pi_{882}^{\left(3\right)}=0.0077$, $\pi_{3073}^{\left(3\right)}=0.0141$.

## Supporting information

S1 File Transition counts. (XLS)
